# Lipid peroxidation and the subsequent cell death transmitting from ferroptotic cells to neighboring cells

**DOI:** 10.1038/s41419-021-03613-y

**Published:** 2021-03-29

**Authors:** Hironari Nishizawa, Mitsuyo Matsumoto, Guan Chen, Yusho Ishii, Keisuke Tada, Masafumi Onodera, Hiroki Kato, Akihiko Muto, Kozo Tanaka, Kazuhiko Igarashi

**Affiliations:** 1 Department of Biochemistry, Tohoku University Graduate School of Medicine, Sendai, 980-8575 Japan; 2 Center for Regulatory Epigenome and Diseases, Tohoku University Graduate School of Medicine, Sendai, 980-8575 Japan; 3 Department of Molecular Oncology, Institute of Development, Aging and Cancer, Tohoku University, Sendai, 980-8575 Japan; 4 Department of Hematology and Rheumatology, Tohoku University Graduate School of Medicine, Sendai, 980-8575 Japan; 5 Department of Medicine, Division of Rheumatology, Lowance Center for Human Immunology, Emory University, Atlanta, GA USA; 6 Department of Pediatric Surgery, Tohoku University Graduate School of Medicine, Sendai, 980-8575 Japan; 7 Department of Human Genetics, National Research Institute for Child Health and Development, Tokyo, 157-8535 Japan

**Keywords:** Cell death, Autophagy

## Abstract

Ferroptosis regulated cell death due to the iron-dependent accumulation of lipid peroxide. Ferroptosis is known to constitute the pathology of ischemic diseases, neurodegenerative diseases, and steatohepatitis and also works as a suppressing mechanism against cancer. However, how ferroptotic cells affect surrounding cells remains elusive. We herein report the transfer phenomenon of lipid peroxidation and cell death from ferroptotic cells to nearby cells that are not exposed to ferroptotic inducers (FINs). While primary mouse embryonic fibroblasts (MEFs) and NIH3T3 cells contained senescence-associated β-galactosidase (SA-β-gal)-positive cells, they were decreased upon induction of ferroptosis with FINs. The SA-β-gal decrease was inhibited by ferroptotic inhibitors and knockdown of *Atg7*, pointing to the involvement of lipid peroxidation and activated autophagosome formation during ferroptosis. A transfer of cell culture medium of cells treated with FINs, type 1 or 2, caused the reduction in SA-β-gal-positive cells in recipient cells that had not been exposed to FINs. Real-time imaging of Kusabira Orange-marked reporter MEFs cocultured with ferroptotic cells showed the generation of lipid peroxide and deaths of the reporter cells. These results indicate that lipid peroxidation and its aftereffects propagate from ferroptotic cells to surrounding cells, even when the surrounding cells are not exposed to FINs. Ferroptotic cells are not merely dying cells but also work as signal transmitters inducing a chain of further ferroptosis.

## Introduction

The notion that dead cells are not only targeted for removal but also important signal transmitters, sending signaling molecules to the surrounding cells and tissues, has recently been attracting attention^1–4^. Apoptotic cells secrete various substances that modulate proliferation^5,6^, regeneration^7,8^, and inflammation/immunity^9,10^. These observations suggest that signal transduction from cells undergoing regulated cell death processes plays important roles in the maintenance of homeostasis of cells and tissues. However, little is known about the signal transduction from nonapoptotic cell death processes, including ferroptosis, necroptosis, and pyroptosis.

Ferroptosis is iron-dependent-regulated necrosis-like cell death^11^. In ferroptosis, the Fenton reaction catalyzed by intracellular labile iron causes lipid peroxidation and hydroxyl-radical generation, leading to cell death^11–13^. This lipid peroxidation is considered the essential cause of ferroptosis. Ferroptosis has been reported to cause ischemic disease in the heart^14–17^, kidney^18,19^, liver^19^, intestine^20^, and brain^21^. In ischemic diseases, the pathological lesion gradually extends even without complete obliteration^22–24^. While repeated ischemic attacks are considered the cause of this extension of the affected area, it is also possible that pathological lesions slowly spread and progress due to lipid peroxide derived from dead cells, including ferroptotic cells.

Some previous works showed the spreading of lipid peroxide and cell deaths during ferroptosis in cultured cells, renal tubules, and zebrafish^18,25–27^. These studies predict the propagating phenomenon of ferroptosis through signaling substances, with reference to the spreading pattern of cell deaths on a dish^25,26^ or the migration of leukocytes in tissues^18,27^. However, the putative spreading of ferroptosis might still require a direct exposure to the FINs used inasmuch as all of the cells were exposed to FINs. For example, exposure to FINs might increase the sensitivity of the cells to propagating signals from ferroptotic cells. In addition, although the recent report shows that ferroptosis caused by type 1 FINs, inhibitors of system Xc cysteine/glutamine antiporter system, propagates like waves^26^, it has not yet been clarified whether a similar propagation occurs by type 2 FINs, inhibitors of glutathione peroxidase 4 (GPX4)^26^.

We tried to further investigate the signaling effect of the secretome from ferroptotic cells induced by either type 1 or type 2 FINs. In particular, we employed conditioned media of ferroptotic cells and coculture of genetically Kusabira orange (KuO) marked cells^28^ to examine conclusively whether lipid peroxidation and cell death could also be spread. We unexpectedly found that cells with the activity of senescence-associated β-galactosidase (SA-β-gal), a classical marker of aging and senescence^29^, were decreased under ferroptotic processes. By using SA-β-gal as an indicator for the progression of ferroptosis in this study, we verified that lipid peroxidation and subsequent cell deaths were propagated from ferroptotic cells to other cells in their vicinity, even when the neighboring cells are not directly exposed to type 1 or 2 FINs.

## Materials and methods

### Mice

The generation of *Bach1*^−/−^ mice on the C57BL/6J background was described previously^30^. The generation of KuO mice on the C57BL/6J background was described previously^28^. These mice were bred at the animal facility of Tohoku University. Mice were housed under specific pathogen-free conditions. Mice were euthanized by cervical dislocation under anesthetic inhalation overdose with isoflurane before anatomy. All experiments performed in this study were approved by the Institutional Animal Care and Use Committee of the Tohoku University Environmental & Safety Committee.

### Isolation of MEFs and cell culture

MEFs were derived from 13.5-day-old embryos of wild-type (WT), *Bach1*^−/−^, or KuO mice. Following removal of the head and organs, embryos were rinsed with PBS (Nissui, Tokyo, Japan), minced, and digested with trypsin (0.05% (v/v) solution containing 0.53 mM EDTA) (Gibco, Carlsbad, CA, USA) and 1.8 mg/ml DNase I (Roche, Basel, Switzerland) in PBS and incubated for 60 min at 37 °C. Trypsin was inactivated by the addition of DMEM with high glucose (Gibco) containing 10% (v/v) fetal bovine serum (FBS) (Sigma-Aldrich, St. Louis, MO, USA), 1× MEM nonessential amino acids (Gibco), and 0.1 mM 2-mercaptoethanol (Sigma-Aldrich). MEFs from a single embryo were plated into a 100-mm-diameter culture dish and incubated at 37 °C in 3% oxygen (1st passage: P1). MEFs from embryos of the same sex littermates were mixed at the 2nd passage (P2) and stocked. MEFs were maintained at 37 °C in culture medium (DMEM (Gibco) with containing 10% FBS (Sigma-Aldrich), 1× MEM nonessential amino acids (Gibco), penicillin/streptomycin (100 U/ml and 100 µg/ml each) (Gibco), and 0.1 mM 2-mercaptoethanol (Sigma-Aldrich)) under 3% oxygen. MEFs were cultured under 20% oxygen after experiments started. The number of passages was recorded for each lot of MEFs. MEFs of 5th–11th passage were used for all experiments. NIH3T3 cells were maintained at 37 °C in culture medium (DMEM with low glucose (Sigma-Aldrich) containing 10% FBS (Sigma-Aldrich), and penicillin/streptomycin (100 U/ml and 100 µg/ml each) (Gibco)) under 20% oxygen for experiments.

### Reagents

Erastin, (1S, 3R)-RSL3 (RSL3), dimethylsulfoxide (DMSO), α-tocopherol (α-Toc), Ferrostatin-1 (Fer-1), chloroquine, and RNase A were purchased from Sigma-Aldrich. MG132 was purchased from Calbiochem (San Diego, CA, USA). Trypsin was purchased from GL Science (Fukushima, Japan).

### RNA interference

All siRNAs (siControl: Stealth RNAiTM siRNA Negative Control, Med GC, siAtg7 #1: MSS232488, siAtg7 #2: MSS292731) were obtained from Invitrogen (Carlsbad, CA, USA). Sequences of the siRNAs are described in Supplementary Table S1. MEFs were transfected with siRNAs using Lipofectamine RNAiMAX (Invitrogen) or Amaxa Nucleofector II (Lonza, Basel, Switzerland) and MF 1 Nucleofector kit (Lonza) according to the manufacturer’s protocols. After transfection, MEFs were passaged to dishes or culture plates with culture medium.

### Western blotting

Cells were trypsinized, pelleted, and washed twice in PBS. Cells were lysed by heating for 5 min in SDS sample buffer (62.5 mM Tris-HCl (pH = 6.8), 1% (v/v) 2-mercaptoethanol, 1% (w/v) sodium dodecyl sulfate, SDS, 10% (w/v) glycerol, and 0.02% (w/v) bromophenol blue, BPB). Lysates were resolved on 7.5–10% SDS-PAGE gels and transferred to PVDF membranes (Millipore, Billerica, MA, USA). The membranes were blocked for 30 min in blocking buffer (5% bovine serum albumin (Sigma-Aldrich) in T-TBS buffer (0.05% Tween 20 (Sigma-Aldrich) in TBS (tris-buffered saline))) and subsequently incubated with the primary antibodies in T-TBS buffer overnight at 4 °C. The antibody for LC3B (2775) was purchased from Cell Signaling Technology, Inc. (Beverly, MA, USA). The antibody for detection of β-actin (GTX109639) was purchased from GeneTex (Irvine, CA, USA). The antibody for GAPDH (ab8245) was purchased from Abcam (Cambridge, UK). For the quantification of signals, all samples to be compared were run on the same gel. Bands were quantified using ImageJ^31,32^. All bands to be compared were quantified on the same image and were within the linear range of detection of the software.

### Quantitative PCR with reverse transcription

The total RNA was purified with RNeasy plus micro kit or RNeasy plus mini kit (Qiagen, Hilden, Germany). Complementary DNA was synthesized by SuperScript III First-Strand Synthesis System (Invitrogen) or High Capacity cDNA Reverse Transcription Kits (Applied Biosystems, Foster City, CA, USA). Quantitative PCR was performed using LightCycler Fast Start DNA Master SYBR Green I, and LightCycler nano (Roche) or LightCycler 96 (Roche). mRNA transcript abundance was normalized to that of *Actb*. Sequences of the qPCR primers are described in Supplementary Table S2.

### Administration of erastin, RSL3, MG132, and chloroquine, and replacement of culture supernatant

Before administration of erastin, RSL3, MG132, or chloroquine, the medium was changed to the experimental medium (culture medium without 2-mercaptoethanol and penicillin/streptomycin) after washing once with PBS. Erastin, RSL3, and MG132 were dissolved in DMSO and administered to the experimental medium. The concentration of DMSO was adjusted among each sample. Chloroquine was dissolved in water and administered to the experimental medium.

In Figs. 3A, F, 4A, 5A, 6A and Supplementary Figs. S6A, D, S7C, S8A, F, experimental medium was changed after washing once with PBS to remove erastin. In Figs. 5A and 6A, KuO MEFs were added after changing the medium.

### Cell death assessment by flow cytometry

Propidium iodide (PI) and annexin V staining were used for the assessment of cell death. In Fig. 5A, 4′,6-diamidino-2-phenylindole (DAPI) was used instead of PI. APC-Annexin V was purchased from Becton, Dickinson and Company (BD) (Franklin Lakes, NJ, USA). MEFs or NIH3T3 cells were stained by APC-Annexin V according to the manufacturer’s protocols. PI was added (1 µg/mL) before flow cytometry. The MEFs or NIH3T3 cells were sorted with a FACS Aria II (BD) or a FACS Verse (BD), and analyzed by FlowJo software (Tree Star, Ashland, OR, USA). Cells that were positive for either or both of annexin V and PI were assessed as dead cells. Conversely, cells that were negative for both annexin V and PI were assessed as alive cells. The gating strategy for assessing alive or dead cells is shown in Supplementary Figs. S2A, B, and S10.

### Detection of SA-β-gal, lipid peroxide, and labile iron

To detect SA-β-gal, lipid peroxide, and mitochondrial labile iron, Cellular Senescence Detection Kit-SPIDER-β Gal (Wako, Osaka, Japan), Quantitative Cellular Senescence Assay (Cell Biolabs, Inc. San Diego, CA, USA), Liperfluo (Dojindo, Kumamoto, Japan)^33,34^, and a fluorophore Mito-FerroGreen (Dojindo)^35^ were used according to the manufacturer’s protocol. For Quantitative Cellular Senescence Assay and Liperfluo, X-gal or Liperfluo was directly administered to experimental medium, including MEFs, respectively 4 h and 1 h before analysis. For Mito-FerroGreen, MEFs were washed three times with Hank’s balanced salt solution (HBSS) (Gibco) to remove the residual medium. Then MEFs were treated with 5 µM Mito-FerroGreen in HBSS at 37 °C for 30 min. After incubation, cells were washed twice with HBSS before analysis. In all of these analyses, alive cells were the target of analysis. The MEFs were sorted with a FACS Aria II (BD) or a FACS Verse (BD), and analyzed by FlowJo software (Tree Star). The gating strategy for alive cells is shown in Supplementary Fig. S2A.

### Cell imaging of lipid peroxide and cell death

MEFs were plated onto 12-well cell culture plates (FALCON, New York, NY, USA). Lipid peroxide was stained by Liperfluo (Dojindo)^33,34^ according to the manufacturer’s protocol. Liperfluo was added to the experimental medium, and MEFs were incubated at 37 °C for an hour. MEFs were observed by fluorescent microscope (CTR6500 HS, Leica Camera, Wetzlar, Germany). The brightness and contrast of pictures in Fig. 5B and Supplementary Fig. S9B, E were adjusted uniformly throughout each picture by Photoshop software version 21.1.3 (Adobe, San Jose, CA, USA). Within each subfigure, the method and degree of adjustment were completely the same. The original pictures before adjustment were shown in Supplementary Fig. S11 and S12.

Cell deaths were assessed by FITC-Annexin V (BD) according to the manufacturer’s protocol in Fig. 6. WT MEFs were plated onto 12-well cell culture plates (FALCON). Before staining, the experimental medium was exchanged to Annexin V binding buffer (BD), including 10% (v/v) FBS. FITC-Annexin V was added to the buffer with KuO MEFs, which were applied to WT MEFs and incubated at 37 °C for 16 h. One hour after adding annexin V and KuO MEFs, serial photography was performed every 10 min for 15h with a fluorescent microscope (Celldiscover7, Carl ZEISS, Oberkochen, Germany) using a ×20 0.70 NA Plan Apochromat lens. One optical channel and two fluorescent (Red: KuO, Green: FITC-Annexin V) channels were used. The brightness of the optical oblique contrast channel of pictures in Fig. 6B was adjusted uniformly throughout each picture by ImageJ and Photoshop software version 21.1.3. The brightness and contrast of fluorescent channels (Red and Green) were not modified. Figure 6C and Supplementary Movies S1A–F are the raw original images.

### RNA-seq and gene set enrichment analysis (GSEA)

We used RNA-seq data of MEFs exposed to erastin from GEO (Gene Expression Omnibus) dataset GSE131444 deposited for our previous report^17^. We used Gene Set Enrichment Analysis (GSEA) to interpret gene expression pattern^36^. Gene sets used in this study, GO_REGULATION_OF_AUTOPHAGY, GO_REGULATION_OF_AUTOPHAGY_OF_MITOCHONDRION, and GO_AUTOPHAGOSOME_MATURATION, were obtained from the Broad Institute.

### Statistics

For all experiments, differences of datasets were considered statistically significant when *P* values were lower than 0.05. Statistical comparisons were performed using the two-sided *t* test in the comparison between the two groups, and one- or two-way ANOVA followed by Tukey’s test in comparison among multiple groups. For the *t* test, Student’s *t* test was used when the standard deviation (SD) of the groups was not significantly different by *f* test. Welch’s *t* test was used when the SD of the groups was significantly different by *f* test. Pearson’s method was used in the correlation analysis. For calculations of significance in GSEA, these were performed as implemented by the GSEA software^36^.

## Results

### SA-β-gal-positive cells are transiently reduced in response to the induction of ferroptosis

**Fig. 1:**
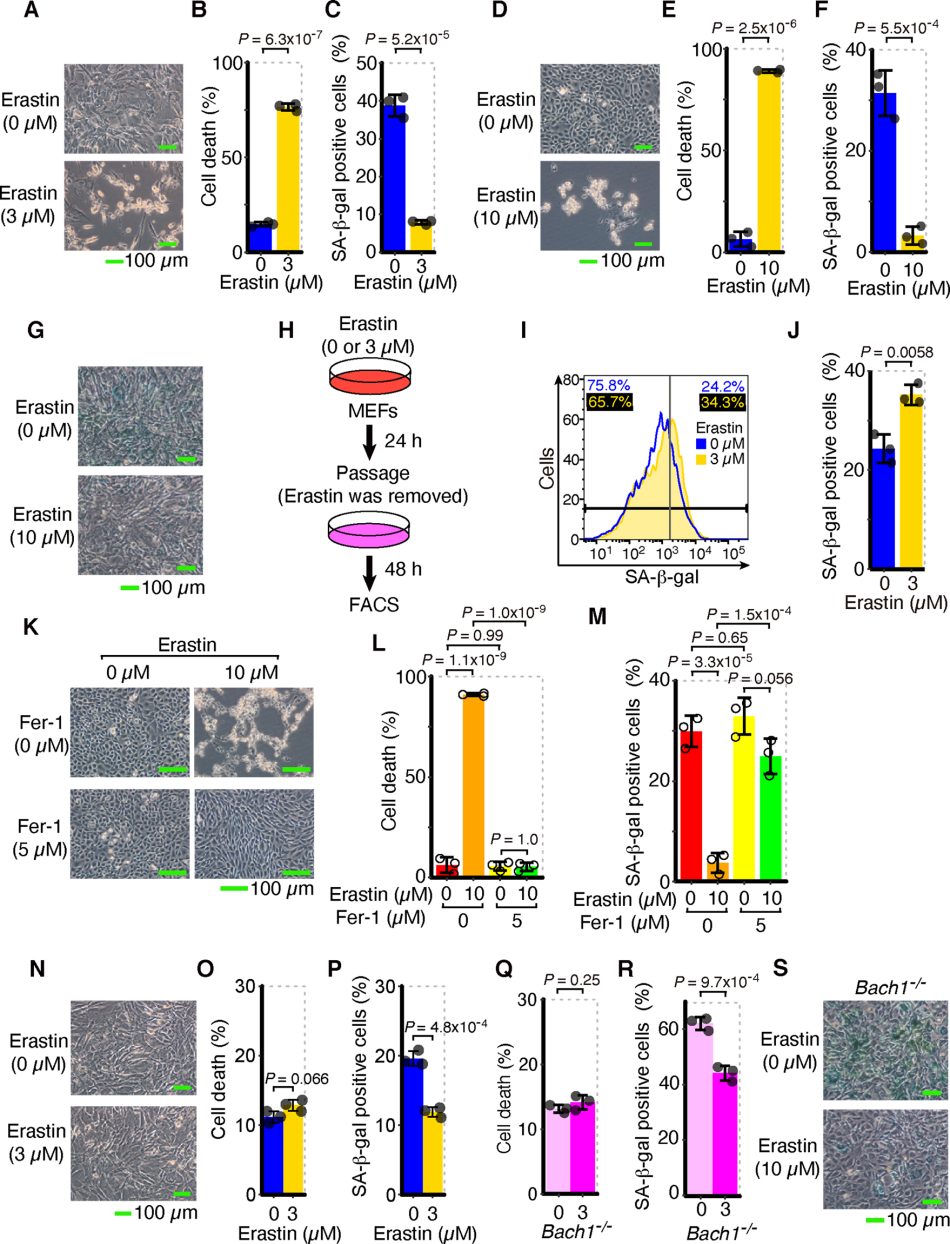
SA-β-gal-positive cells are transiently reduced in response to lipid peroxidation during ferroptosis. **A**–**C** WT MEFs were exposed to erastin for 24 h. **A** Optical microscope image. **B**, **C** Quantification of dead cells (**B**) and SA-β-gal-positive cells (**C**) by flow cytometer. **D**–**F** NIH3T3 cells were exposed to erastin for 24 h. **D** Optical microscope image. **E**, **F** Quantification of dead cells (**E**) and SA-β-gal-positive cells (**F**) by flow cytometer. **G** WT MEFs were exposed to erastin for 24 h. Optical microscope image after SA-β-gal staining. **H**–**J** After WT MEFs had been exposed to erastin for 24 h, they were passaged in culture medium (not including erastin) and cultured for 48 h. **H** Experimental outline. **I** Representative data of histogram of SA-β-gal fluorescence by flow cytometer. Values on the upper left and upper right represent respectively the percentage of negative and positive cells. The colors correspond to histogram traces. **J** Quantification of SA-β-gal-positive cells by flow cytometer. **K**–**M** NIH3T3 cells were exposed to erastin and Fer-1 for 24 h. **K** Optical microscope image. **L**, **M** Quantification of dead cells (**L**) and SA-β-gal-positive cells (**M**) by flow cytometer. **N**–**P** WT MEFs were exposed to erastin for 12 h. **N** Optical microscope image. **O**, **P** Quantification of dead cells (**O**) and SA-β-gal-positive cells (**P**) by flow cytometer. **Q**–**S** *Bach1*^−/−^ MEFs were exposed to erastin for 24 h. Quantification of dead cells (**Q**) and SA-β-gal-positive cells (**R**) by flow cytometer, and optical microscope image after SA-β-gal staining (**S**). Scale bars in **A**, **D**, **G**, **K**, **N**, and **S** represent 100 µm. **Q** and **R** are representative of three independent experiments. **A**–**P** and **S** are representative of two independent experiments carried out in triplicate. Error bars of **B**, **C**, **E**, **F**, **J**, **L**, **M**, **O**, **P**, **Q**, and **R** represent SD. *P* value of **B**, **C**, **E**, **F**, **J**, **O**, **P**, **Q**, and **R** by two-sided *t* test. *P* value of **L** and **M** by Tukey’s test after two-way ANOVA.

To validate the signaling effect from ferroptotic cells, we needed a sensitive indicator beside the cell death markers. We focused on senescence-associated β-galactosidase (SA-β-gal), a classical senescence marker^29^. Oxidative stress is a major factor affecting cellular senescence^37,38^. We predicted that the SA-β-gal-positive cells might be increased during ferroptosis because lipid peroxidation and oxidative stress forcefully accumulate in ferroptotic cells^11–13^. First, we measured cells with SA-β-gal in mouse embryonic fibroblasts (MEFs) and NIH3T3 cells exposed to erastin, a type 1 FIN^11^. Unexpectedly, SA-β-gal-positive cells were not increased but rather decreased in MEFs and NIH3T3 cells exposed to erastin, in contrast to the increase in cell death (Fig. 1A–G and Supplementary Figs. S1, S2). Although the decrease in SA-β-gal-positive cells was still kept in NIH3T3 cells even 3 days after erastin was removed (Supplementary Fig. S3A–C), it was canceled or rather increased when erastin was removed and the MEFs were recultured for 2 days (Fig. 1H–J and Supplementary Fig. S3D). Therefore, the decrease in SA-β-gal-positive cells in response to erastin can be transient or sustained, depending on cells, and does not reflect suppression of typical cellular senescence.

Next, we examined whether or not the decrease in SA-β-gal-positive cells was canceled by inhibition of ferroptosis. We administered two types of ferroptosis inhibitors, ferrostatin-1 (Fer-1) and α-tocopherol (α-Toc), to NIH3T3 cells or MEFs exposed to erastin. Fer-1 is a specific inhibitor of ferroptosis, that efficiently inhibits lipid peroxidation^11^, and α-Toc is a general reducing agent and also inhibits lipid peroxidation and ferroptosis^12,39^. These inhibitors indeed suppressed cell death by erastin (Fig. 1K, L and Supplementary Fig. S3E, F) and also canceled or alleviated the decrease in SA-β-gal-positive cells (Fig. 1M and Supplementary Fig. S3G), indicating that the decrease in SA-β-gal-positive cells during ferroptosis reflected the progression of ferroptosis.

To rule out the possibility that this reduction in SA-β-gal-positive cells was simply due to the removal of senescent cells by cell death, we analyzed the changes in SA-β-gal staining at an earlier stage of exposure to erastin, when cell death had not yet been induced. We discovered that the reduction in SA-β-gal-positive cells preceded cell death (Fig. 1N–P). These results suggest that the observed decrease in SA-β-gal-positive cells upon ferroptosis is not simply due to the removal of senescent cells by cell death, but is an integrated cellular response of ferroptosis. In addition to wild-type (WT) MEFs, we also examined SA-β-gal staining profiles among *Bach1*^−/−^ MEFs, which are more resistant to ferroptosis and more prone to senescence than WT MEFs^17,40,41^. We confirmed that the reduction in SA-β-gal-positive cells was also observed in *Bach1*^−/−^ MEFs (Fig. 1Q–S and Supplementary Figs. S1D, S3H). In summary, the reduction in SA-β-gal-positive cells during ferroptosis can be detected even before cell death assessed by cell death markers and even in *Bach1*^−/−^ cells that are resistant to ferroptosis. Thus, it was considered highly sensitive and useful in this study to investigate the signaling effect of ferroptotic cells.

### Activation of autophagosome formation in ferroptotic cells leads to a decrease in SA-β-gal staining

**Fig. 2:**
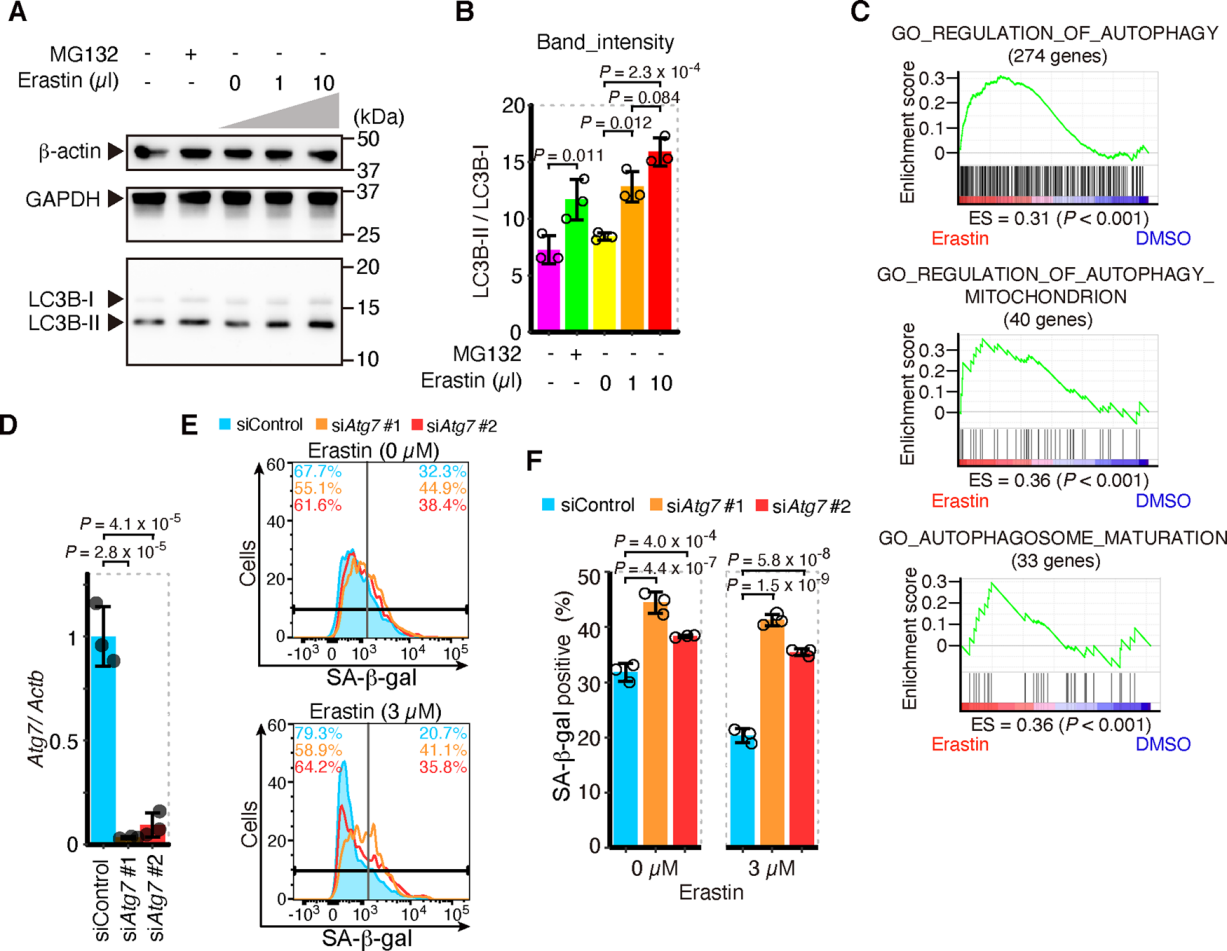
Activation of autophagosome formation in ferroptotic cells leads to a decrease of SA-β-gal staining. **A***,* **B** Western blotting for LC3B, β-actin, and GAPDH of MEFs exposed to erastin or 25 µM MG132 for 12 or 6 h, respectively. **A** Representative image and (**B**) the intensity of bands of LC3B. **C** Gene set enrichment analysis (GSEA) of genes related to autophagy regulation and autophagosome maturation in erastin or DMSO-treated MEF (left and right at the bottom); below plots, enrichment score (ES) and nominal *P* value, as implemented in GSEA. **D**–**F** siRNA was introduced to MEFs with lipofection. After 24 h, erastin was administered to MEFs. After 24 h of administration of erastin, SA-β-gal was assessed. **D** qPCR analysis for *Atg7* mRNA relative to *Actb* mRNA. **E** Representative data of the histogram of SA-β-gal fluorescence by flow cytometer. Values on the upper left and upper right represent, respectively, the percentage of negative and positive cells. The colors correspond to histogram traces. **F** Quantification of SA-β-gal-positive cells by flow cytometer. **D–F** are representative of two independent experiments carried out in triplicate. Error bars of **B**, **D**, and **F** represent SD. *P* value of **B** and **F** by Tukey’s test after two-way ANOVA. *P* value of **D** by Tukey’s test after one-way ANOVA.

We next explored how SA-β-gal-positive cells were decreased upon the induction of ferroptosis. SA-β-gal is reported to be encoded by the same gene as lysosomal β-galactosidase: *Glb1*^42^. We first checked the expression of *Glb1* upon administration of erastin, but the expression was not decreased (Supplementary Fig. S4A). It has been reported that autophagy and lysosomes are activated during ferroptosis^43–47^. We also reconfirmed that autophagosome formation and subsequent lysosomal activity were promoted during ferroptosis (Fig. 2A–C and Supplementary Fig. S4B–E). The protein stability and enzymatic activity of SA-β-gal may be affected by the enhancement of autophagosome formation and lysosomal activity during ferroptosis.

We knocked down the *Atg7* gene in MEFs (Fig. 2D and Supplementary Fig. S5E) to prevent the formation of autophagosomes. The knockdown of *Atg7* reduced cell deaths by erastin (Supplementary Fig. S5A, B). While intracellular labile iron is elevated by ferritinophagy in ferroptosis^17,43^, the elevation of labile iron in MEFs by erastin was also suppressed (Supplementary Fig. S5C, D). These results indicate that the formation of autophagosome and subsequent ferritinophagy was prevented by the knockdown of *Atg7*. We found that knockdown of *Atg7* elevates SA-β-gal-positive cells, both with and without erastin exposure (Fig. 2E, F and Supplementary Fig. S5F–H). These results suggest that the activation of autophagosome formation reduces SA-β-gal-positive cells during ferroptosis. On the other hand, the exposure to chloroquine, a lysosomal inhibitor, did not increase the numbers of SA-β-gal-positive cells, but rather decreased them (Supplementary Fig. S4F–K). Therefore, accelerated autophagosome formation appears to be the cause of the decrease in SA-β-gal-positive cells during ferroptosis, independently of lysosomal activation. In any case, the decrease in SA-β-gal-positive cells is suppressed by inhibiting lipid peroxidation and autophagosome formation (Figs. 1M, 2F and Supplementary Figs. S3G, S5H), both of which are essential pathways of ferroptosis, and thus may be a useful indicator of the ferroptotic response in this study.

### The reduction in SA-β-gal-positive cells propagates from ferroptotic cells to other cells

**Fig. 3:**
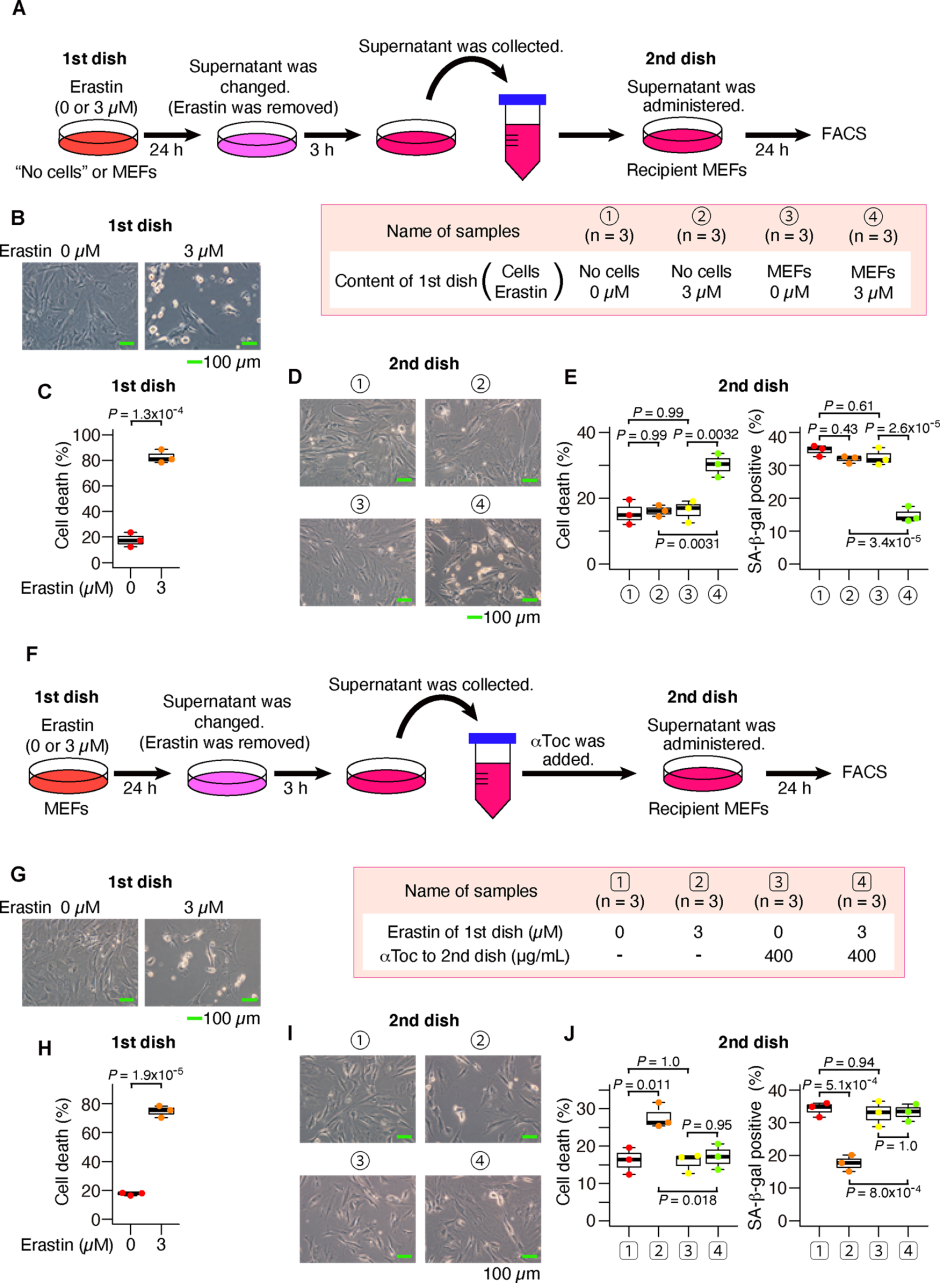
The supernatant from ferroptotic cells increased cell death and decreased SA-β-gal in recipient cells. **A**–**C** After MEFs had been exposed to erastin for 24 h, the supernatant medium was exchanged (erastin was removed). After 3 h, the supernatant medium was collected and administered to recipient MEFs. **A** Experimental outline. **B**, **C** These are data of donor MEFs. **B** Optical microscope image. **C** Quantification of dead cells by flow cytometer. **D**, **E** These are the data of recipient MEFs. **D** Optical microscope image. **E** Quantification of dead cells and SA-β-gal-positive cells by flow cytometer. **F**–**J** α-Toc was added to the conditioned medium prepared as in **A**. **F** Experimental outline. **G**, **H** These are data of donor MEFs. **G** Optical microscope image. **H** Quantification of dead cells by flow cytometer. **I**, **J** These are the data of recipient MEFs. **I** Optical microscope image. **J** Quantification of dead cells and SA-β-gal-positive cells by flow cytometer. Scale bars in **B**, **D**, **G**, and **I** represent 100 µm. The box-and-whisker plots of **C**, **E**, **H**, and **J** show the 25th and 75th percentile quartiles and median values (center black line) and maximum and minimum values of the data. *P* value of **C** and **H** by two-sided *t* test. *P* value of **E** and **J** by Tukey’s test after two-way ANOVA.

The decrease in SA-β-gal-positive cells in ferroptotic cells may provide a sensitive experimental system for examining the intercellular signaling effects of ferroptosis. To investigate the effects of ferroptotic cells on the surrounding cells, we collected supernatant medium from ferroptotic cells and administered it to recipient cells. As indicated in Fig. 3A, we exposed MEFs to erastin, removed erastin by exchanging the supernatant medium to exclude the direct effects of erastin, and continued the culture. We obtained a supernatant medium containing secreted substances from ferroptotic cells but containing no erastin. When we administered the supernatant medium to recipient MEFs, the cell death was not increased, but SA-β-gal-positive cells were decreased (Supplementary Figs. S6A–C and S7A). Furthermore, when the donor cells were cultured at lower cell density, not only a decrease in SA-β-gal-positive cells but also an increase in cell death was observed in recipient cells (Fig. 3A–E). This decrease in SA-β-gal-positive cells and the increase in cell death were not observed in the supernatant medium from the dishes containing only erastin without cells (Fig. 3A–E and Supplementary Fig. S6A–C). Given these results, it was considered that the decrease in SA-β-gal-positive cells was not due to the potential remaining erastin in the dishes, but was instead caused by the effect of a substance secreted by ferroptotic cells.

We, therefore, attempted to characterize this secreted substance. Considering that the secretory substance may be lipid peroxide from ferroptotic cells, we added α-Toc to the supernatant medium (Fig. 3F). In this protocol, α-Toc suppressed cell death and the decrease in SA-β-gal-positive cells in recipient MEFs (Fig. 3F–J and Supplementary Figs. S6D–F, S7B), indicating that lipid peroxidation was propagated in the secreted substances from ferroptotic cells. Since other types of molecules, such as microRNAs and proteins, may be secreted by ferroptotic cells and cause a reduction in SA-β-gal-positive cells, we examined whether or not RNase or protease suppressed the decrease in SA-β-gal-positive cells caused by the supernatant of ferroptotic cells (Supplementary Fig. S7C). Unlike α-Toc, these treatments did not reverse the reducing effect of SA-β-gal-positive cells by the ferroptotic supernatant (Supplementary Fig. S7D, E). Although the identity of the secretory substances needs to be verified in the future, the above results strongly suggest that they are lipid peroxide.

**Fig. 4:**
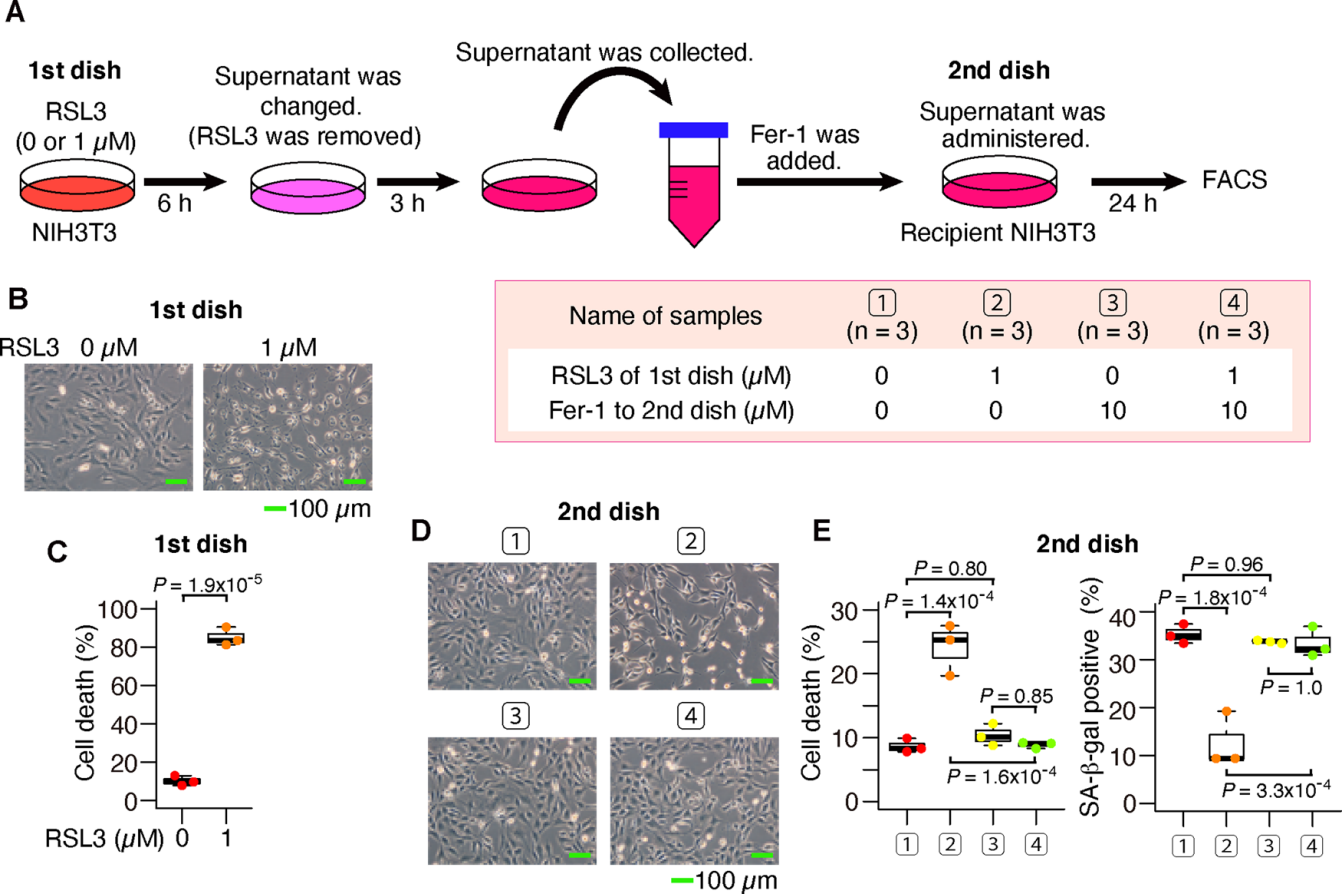
The supernatant from RSL3-induced ferroptotic cells also increased cell death and decreased SA-β-gal in recipient cells. **A**–**D** After NIH3T3 cells had been exposed to (1S, 3R)-RSL3 for 6 h, the supernatant medium was exchanged (RSL3 was removed). After 3 h, the supernatant medium was collected and administered to recipient NIH3T3 cells. **A** Experimental outline. **B**, **C** These are data of donor NIH3T3 cells. **B** Optical microscope image. **C** Quantification of dead cells by flow cytometer. **D**, **E** These are data of recipient NIH3T3 cells. **D** Optical microscope image. **E** Quantification of dead cells and SA-β-gal-positive cells by flow cytometer. Scale bars in **B** and **D** represent 100 µm. The box-and-whisker plots of **C** and **E** show the 25th and 75th percentile quartiles and median values (center black line) and maximum and minimum values of the data. *P* value of **C** by two-sided *t* test. *P* value of **E** by Tukey’s test after two-way ANOVA.

For further validation, we replaced MEFs with NIH3T3 cells, the ferroptosis inducer with (1S, 3R)-RSL3 (RSL3), a type 2 FIN, and the inhibitor of lipid peroxidation with Fer-1 to confirm the propagation of cell death and decrease in SA-β-gal-positive cells (Fig. 4A). The supernatant medium from cells exposed to RSL3 also had an effect of decreasing SA-β-gal-positive cells and increasing cell deaths, which was inhibited by Fer-1 (Fig. 4B–E and Supplementary Figs. S7F, S8A–E). We obtained similar results with MEFs (Supplementary Fig. S8F–J). These results indicate that the spreading of ferroptotic reaction is a universal phenomenon of ferroptosis, which is not limited to only type 1 FINs.

### Lipid peroxidation and cell death also propagate from ferroptotic cells to other cells

**Fig. 5:**
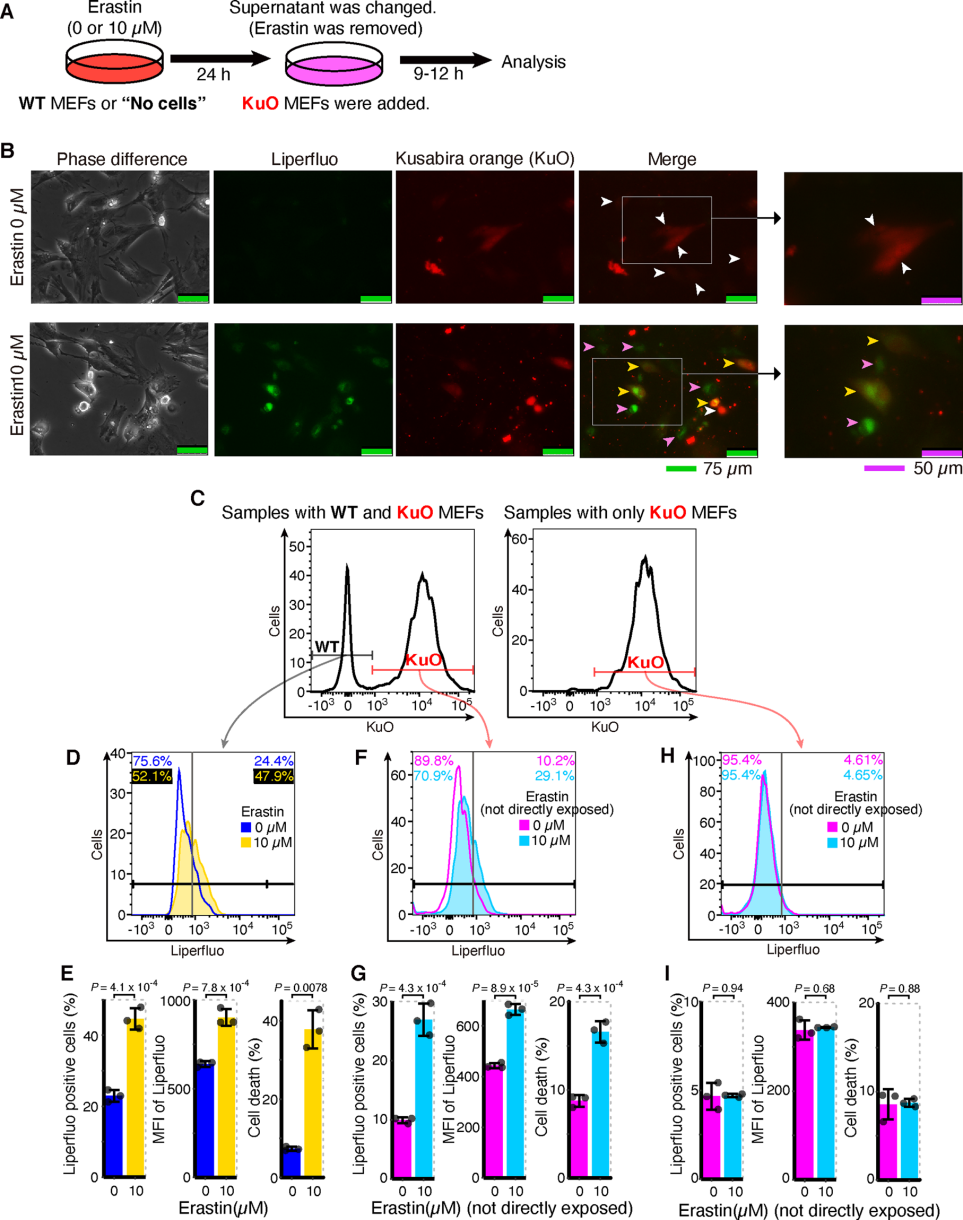
Lipid peroxidation and cell deaths propagated from ferroptotic cells to surrounding cells. **A**–**G** After WT MEFs had been exposed to erastin, the supernatant medium was exchanged (erastin was removed). At that time, Kusabira Orange (KuO) mice-derived MEFs were added. After 9 h from adding KuO MEFs, MEFs were analyzed by a fluorescent microscope. After 12 h from adding KuO MEFs, MEFs were analyzed by a flow cytometer. **A** Experimental outline. **B** Optical and fluorescent microscope image after Liperfluo staining. White arrowheads: KuO MEFs without lipid peroxidation. Pink arrowheads: WT MEFs with lipid peroxidation. Yellow arrowheads: KuO MEFs with lipid oxidation. **C** Representative data of the histogram of KuO fluorescence by flow cytometer to distinguish KuO MEFs from WT MEFs. **D**, **F** Representative data of the histogram of Liperfluo fluorescence in WT MEFs (**D**) or KuO MEFs (**F**) by flow cytometer. Values on the upper left and upper right represent, respectively, the percentage of negative and positive cells. The colors correspond to histogram traces. **E**, **G** Quantification of Liperfluo-positive cells, Liperfluo MFI, and cell deaths in WT MEFs (**E**) or KuO MEFs (**G**) by flow cytometer. **H**, **I** Erastin was added to dishes without cells. After 24 h, the supernatant medium was exchanged (erastin was removed). At that time, KuO MEFs were added. After 12 h from adding KuO MEFs, MEFs were analyzed by a flow cytometer. **H** Representative data of the histogram of Liperfluo fluorescence in KuO MEFs as above. **I** Quantification of Liperfluo-positive cells, Liperfluo MFI, and cell deaths in KuO MEFs by flow cytometer. Light-green scale bars in **B** represent 75 µm. Purple scale bars in **B** represent 50 µm. All data are representative of two independent experiments. Error bars of **E**, **G**, and **I** represent SD. *P* value of **E**, **G**, and **I** by unpaired two-sided *t* test. MFI mean fluorescence intensity.

Next, we sought to determine whether or not lipid peroxidation and cell death propagate from ferroptotic cells to other cells using the fluorescent reporter Liperfluo^33,34^ and MEFs prepared from transgenic mice expressing the fluorescent protein Kusabira Orange (KuO)^28^. First, we confirmed that lipid peroxidation was increased in correspondence with cell death in MEFs exposed to erastin (Supplementary Fig. S9A–D). We then administered erastin to WT MEFs and replaced the supernatant medium to remove erastin, seeding KuO MEFs there and coculturing these cells with KuO MEFs to examine whether or not the increased lipid peroxidation in WT MEFs also affects KuO MEFs (Fig. 5A). Using fluorescent microscopy, we confirmed that lipid peroxidation was indeed increased in KuO MEFs that had not been directly exposed to erastin (Fig. 5B and Supplementary Fig. S9E). A flow cytometry analysis further confirmed that an increase in lipid peroxidation was observed not only in WT MEFs but also in cocultured KuO MEFs (Fig. 5C–G). In addition to the increase in lipid peroxidation, cell death was also increased in KuO MEFs, as well as in WT MEFs (Fig. 5C–G and Supplementary Fig. S10A–C). In dishes without WT MEFs, we noted no increase in lipid peroxidation or cell death in KuO MEFs added after the replacement of the erastin-containing medium (Fig. 5C, H, I and Supplementary Fig. S10D, E). Given these results, we inferred that the erastin-induced lipid peroxidation in WT MEFs was transmitted to cocultured KuO MEFs. This indicates that lipid peroxidation in ferroptotic cells propagates to surrounding cells. Our experimental results suggest that if ferroptosis arises in some cells, the signal then spreads to the surrounding cells in the population and causes a ferroptotic chain reaction via the propagation of lipid peroxidation.

**Fig. 6:**
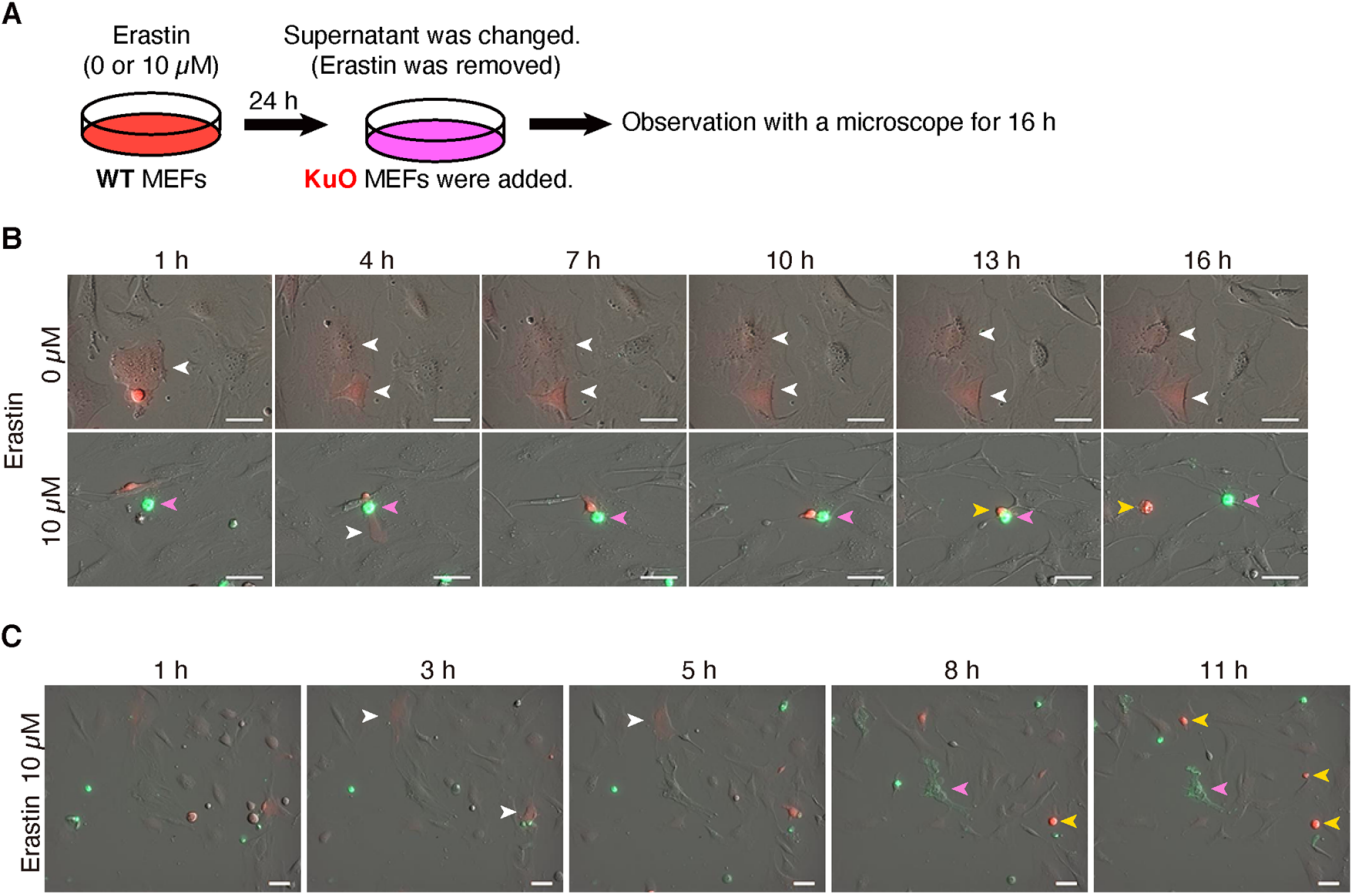
Cell deaths propagated from ferroptotic cells to other cells. **A**–**C** After WT MEFs had been exposed to erastin, the supernatant medium was exchanged (erastin was removed). At that time, KuO mice-derived MEFs were added and cells were observed by fluorescent microscope for 16 h. **A** Experimental outline. **B**, **C** Merged image of optical and fluorescent (Red: KuO, Green: FITC-Annexin V) channel. **B**, **C** are from separate experiments. White arrowheads: alive KuO MEFs. Pink arrowheads: dead WT MEFs. Yellow arrowheads: dead KuO MEFs. Scale bars represent 50 µm.

Finally, we sought to capture the sequence of cell death propagating from WT MEF to KuO MEFs by serial photography. Similar to Fig. 5A, we exposed WT MEFs to erastin for 24 h and exchanged the supernatant medium, subsequently seeding KuO MEFs there (Fig. 6A). We assessed the cell death with Annexin V. In the wells receiving erastin, the KuO MEFs not exposed to erastin also died in the vicinity of dead WT MEFs (Fig. 6B and Supplementary Movie S1A–E). Furthermore, we captured the WT MEFs dying due to erastin-exposure stress and observed that KuO MEFs around the dead WT MEFs also died a short while later (Fig. 6C and Supplementary Movie S1F). Combined with the findings in Fig. 5, these results showed that a chain reaction of cell deaths occurred in the surrounding cells due to the propagation of lipid peroxidation from ferroptotic cells.

## Discussion

We showed that lipid peroxidation in ferroptotic cells is transmitted to other cells in a paracrine manner. This suggests that once ferroptosis occurs, the effect propagates to the surrounding cells, and ferroptosis events continue in a chain. Whether or not a similar mechanism occurs in vivo should be verified. If we consider tissue fluid or blood as culture medium, a similar phenomenon could indeed occur in vivo. In the previous reports, it has been shown that leukocyte migration is triggered by signals from ferroptotic cells^18,27^, and our study complements the contents of these papers. In cases of stroke, where the pathology is associated with ferroptosis^21^, there is a pathological lesion called a “penumbra” around the infarct lesion that is not exposed to complete ischemia^48,49^. If ferroptosis occurs in the infarct area of stroke, the “penumbra” may expand along with the spread of lipid peroxidation, as shown in this study. In addition to stroke, other ferroptosis-related diseases, such as ischemic heart disease^14–17^, neurodegenerative disease^50,51^, and steatohepatitis^52,53^, may have the same underlying mechanism. Therefore, it will be necessary to consider the propagating effects of lipid peroxidation from ferroptotic cells in order to better understand the associated pathophysiology. Conversely, the propagation of lipid peroxidation from ferroptotic cells can be a prospective target for exploring new ways to treat these diseases.

We found that the numbers of SA-β-gal-positive cells were reduced by lipid peroxidation in ferroptosis. This reduction was transient and did not reflect the suppression of cellular senescence per se or the demise of SA-β-gal-positive cells (Fig. 1H–J). The decrease in SA-β-gal-positive cells was canceled by inhibiting lipid peroxidation and autophagosome formation (Figs. 1K–M, 2E, F and Supplementary Fig. S3E–G). Because lipid peroxidation and autophagy are promoted in ferroptotic cells (Fig. 2A–C and Supplementary Fig. S9B–D), SA-β-gal may be removed by autophagy in cells undergoing ferroptosis.

It was suggested in this study that ferroptotic cells act as signal transmitters. The propagation of lipid peroxidation and cell death in a culture dish has already been reported by the elegant prediction based on the spreading pattern of cell deaths^25,26^. Our findings confirmed and further complemented those previous studies. In particular, we were able to show that transfer of culture supernatants and coculture propagated the ferroptotic response, suggesting that the secretome from ferroptotic cells is important for the propagation of lipid peroxidation and cell death. In addition, we demonstrated that a signaling effect from ferroptotic cells was observed even in the case of type 2 FINs (Fig. 4A–E). This point was elusive in the previous study^26^. In the case of type 2 FINs, the effect of the drug itself is probably stronger than the propagation effect of lipid peroxidation; thus, only observing the spreading pattern of cell death in the dish would not have been enough to determine whether or not there was a signal effect from ferroptotic cells. Therefore, combining the recent work^26^ with our findings, we conclude that ferroptosis can propagate to neighboring cells depending on signal substances.

Although cell death was increased in the recipient cells cocultured with ferroptotic cells (Figs. 5E, G, I, 6B, C and Supplementary Movie S1A–F), an increase in cell death was not observed following the transfer of supernatant medium from ferroptotic cells unless the donor and recipient cell density was lowered (Supplementary Figs. S6A–C, S8A–E). These observations suggest that the signal substances may be unstable. This study suggested that lipid peroxides were signal substances (Figs. 3F–J, 4, 5 and Supplementary Fig. S7C–E). They may be unstable lipid mediators, like prostanoids in the arachidonic acid pathway^54^. Since it has been reported that various substances are secreted from apoptotic cells^7–10^, multiple signal substances, not only lipid peroxide, are also considered to be secreted from ferroptotic cells. A further analysis of the secretomes from ferroptotic cells will be important to clarify the significance and roles of ferroptosis in vivo. Given that the biological role of ferroptosis in vivo has not been clarified, except for cancer, a secretome analysis is expected to further elucidate the significance of ferroptosis in the human body.

With respect to cancer, ferroptosis has been repeatedly reported to work as a tumor-suppressive mechanism as well as apoptosis^55–58^, and has been attracting attention as a novel therapeutic method against drug-tolerant cancer cells^59,60^. If the chain of ferroptosis can be reinforced by propagating lipid peroxidation, it may work effectively for cancer treatment. Certain types of cancer are reported to be particularly sensitive to lipid peroxidation^61,62^. Therefore, we expect to improve the efficiency of cancer therapy by exploiting the propagation of lipid peroxide. However, lipid peroxide and other mediators generated by cancer cells through ferroptosis may cause cachexia or adverse effects under anticancer therapy. If we can suppress the spread of presumptive ferroptosis mediators to normal tissue in addition to encouraging ferroptosis in cancer tissue, we may be able to prevent adverse effects.

## Supplementary information

Supplementary Figure legends

Supplementary Movie legends

Supplementary Tables

Supplementary Figure S1

Supplementary Figure S2

Supplementary Figure S3

Supplementary Figure S4

Supplementary Figure S5

Supplementary Figure S6

Supplementary Figure S7

Supplementary Figure S8

Supplementary Figure S9

Supplementary Figure S10

Supplementary Figure S11

Supplementary Figure S12

Supplementary Movie S1A

Supplementary Movie S1B

Supplementary Movie S1C

Supplementary Movie S1D

Supplementary Movie S1E

Supplementary Movie S1F

## Data Availability

RNA-Seq analyses discussed in this publication have been deposited in the National Center for Biotechnology Information’s Gene Expression Omnibus and linked sequence read archive files, which are accessible through GEO Series accession no. GSE131444.
