# Supplementary material for: Lipid peroxidation and the subsequent cell death transmitting from ferroptotic cells to neighboring cells
Source: Cell Death Dis. 2021 Mar 29;12(4):332. doi: 10.1038/s41419-021-03613-y (PMC8007748; doi:10.1038/s41419-021-03613-y)
Supplement: Supplementary file 1 — Supplementary Figure legends [file 41419_2021_3613_MOESM1_ESM.docx]

**Supplementary Figure S1. SA-β-galactosidase (SA-β-gal) was reduced in ferroptotic cells.**

*A-C*, Additional data of Fig. 1*A-C*. Cell density is higher than in Fig.1*A-C*. Wild type (WT) mouse embryonic fibroblasts (MEFs) were exposed to erastin for 24 hours (h). (*A*) Optical microscope image. (*B* and *C*) Quantification of dead cells (*B*) and SA-β-gal-positive cells (*C*) by flow cytometer. *D*, WT and *Bach1*^-/-^ MEFs were exposed to erastin for 24 h. Optical microscope image after SA-β-gal staining. Scale bars in *B* and *D* represent 100 µm. White rectangles show the part of Fig. 1*G* and *S*.

**Supplementary Figure S2. Additional data demonstrating flow cytometry gating.**

*A* and *B*, Representative flow cytometry images showing the strategy that was implemented for the sorting of alive cells (*A*). Both propidium iodide (PI) and Annexin V negative cells were judged as alive cells. The other cells were judged as dead cells. SA-β-gal, lysosomal activity, and Liperfluo were assessed in alive cells (*A*). No stained control was used to decide the gate line of SA-β-gal, lysosomal activity, Mito-FerroGreen, and Liperfluo (*B*). Representative data of (*A*) was that of WT MEFs and erastin 0 µM in Fig.S1*B* and *C*. The similar strategy was implemented in Fig. 1*-*4, Fig. S3-9. *C*-*F*, Additional data of Fig. 1*A-F*. A representative flow cytometry image of Fig. 1*B* (*C*) and Fig. 1*E* (*E*). Quantification of SA-β-gal positive cells in not only alive cells but also whole cells of Fig. 1*C* (*D*) and Fig. 1*F* (*F*). From *C* and *E*, more than 1000 cells and 300 cells respectively were analyzed in Fig. 1*C* and *F.* From *D* and *F*, the decrease of SA-β-gal positive cells under the induction of ferroptosis was observed in the analysis of whole cells.

**Supplementary Figure S3. SA-β-gal was reduced in ferroptotic cells.**

*A-C*, After NIH3T3 cells had been exposed to erastin for 12 h, they were passaged in culture medium (not including erastin) and cultured for 72 h. (*A*) Experimental outline. (*B*) Optical microscope image. (*C*) Quantification of SA-β-gal-positive cells by flow cytometer. *D*, Additional data of Fig. 1*H-J*. After WT MEFs had been exposed to erastin for 24 h, they were passaged in culture medium (not including erastin) and cultured for 48 h. (*D*) Optical microscope image. *E-G*, WT MEFs were exposed to erastin and α-Toc for 24 h. (*E*) Optical microscope image. (*F and G*) Quantification of dead cells (*F*) and SA-β-gal positive cells (*G*) by flow cytometer. *H*, Additional data of Fig. 1*Q* and *R*. *Bach1*^-/-^ MEFs were exposed to erastin for 24 h. (*H*) Optical microscope image. Scale bars in *B, D, E,* and *H* represent 100 µm. *A-G* are representative of two independent experiments. *H* is representative of three independent experiments. Error bars of *C, F,* and *G* represent S.D. *p* value of *C* by two-sided *t*-test. *p* value of *F* and *G* by Tukey’s test after two-way ANOVA.

**Supplementary Figure S4. Autophagy-lysosome pathway was activated in ferroptosis. Blocking lysosomal function did not cancel the decrease of SA-β-gal positive cells during ferroptosis.**

(*A*) MEFs were exposed to erastin for 10 h. qPCR analysis for *Glb1* mRNA relative to *Actb* mRNA. *B-E*, WT MEFs were exposed to erastin for 7 h. (*B*) Optical microscope image. (*C*) Quantification of dead cells by flow cytometer. (*D*) Representative data of histogram of the fluorescence from lysosomal intracellular activation by flow cytometer. Numbers on the left and right sides represent respectively the percentage of negative and positive cells. (*E*) Quantification of lysosomal intracellular activity by flow cytometer. Pearson's correlation analysis between concentration of erastin and lysosomal intracellular activity. *F-H*, MEFs were exposed to chloroquine for 24 h. (*F*) Optical microscope image. Quantification of dead cells (*G*) and SA-β-gal positive cells (*H*) by flow cytometer. *I-K*, WT MEFs were exposed to erastin and chloroquine for 30 h. (*I*) Optical microscope image. (*J* and *K*) Quantification of dead cells (*J*) and SA-β-gal positive cells (*K*) by flow cytometer. Scale bars in *B,* *F,* and *I* represent 100 µm. All data are representative of two independent experiments. The box and whisker plots of *C* and *E* show the 25th and 75th percentile quartiles and median values (center black line) and maximum and minimum values of the data. Error bars of *G, H,* *J*, and *K* represent S.D. *p* value of *A, G,* and *H* by Tukey’s test after one-way ANOVA. *p* value of *C* by one-way ANOVA. *p* value of *E* by Pearson's correlation analysis. *p* value of *J* by Tukey’s test after two-way ANOVA. *p* value of *K* by unpaired two-sided *t* test. MFI: Mean fluorescence intensity.

**Supplementary Figure S5. Inhibiting autophagosome formation led to increase of SA-β-gal positive cells.**

*A-D*, Additional data of Fig. 2*D-F*. siRNA for *Atg7* was introduced to MEFs with lipofection. After 24 h, erastin was administered to MEFs. After 24 h from administration of erastin, cell deaths and SA-β-gal was assessed. (*A*) Optical microscope image. (*B*) Quantification of dead cells by flow cytometer. (*C*) Representative data of histogram of SA-β-gal fluorescence by flow cytometer. Numbers on the upper left and upper right represent respectively the percentage of negative and positive cells. The colors correspond to histogram traces. (*D*) Quantification of mitochondrial labile iron by flow cytometer. *E-H*, siRNA for *Atg7* was introduced to MEFs with electroporation. After 48 h from administration of erastin, SA-β-gal was assessed. (*E*) qPCR analysis for *Atg7* mRNA relative to *Actb* mRNA. (*F*) Optical microscope image. (*G*) Representative data of histogram of SA-β-gal fluorescence. Numbers on the upper left and upper right represent respectively the percentage of negative and positive cells. The colors correspond to histogram traces. (*H*) Quantification of SA-β-gal positive cells by flow cytometer. Scale bars in *A* and *F* represent 100 µm. All data are representative of two independent experiments. Error bars of *B, D, E,* and *H* represent S.D. *p* value of *B* and *D* by Tukey’s test after two-way ANOVA. *p* value of *E* and *H* by Tukey’s test after one-way ANOVA.

**Supplementary Figure S6. The supernatant from ferroptotic cells decreased SA-β-gal of recipient cells.**

*A-C*, Additional data of Fig. 3*A-E*. Cell density was higher than in Fig. 3*A-E*. After MEFs had been exposed to erastin for 24 h, the supernatant medium was exchanged (erastin was removed). After 3 h, the supernatant medium was collected and administered to recipient MEFs. (*A*) Experimental outline. (*B*) Optical microscope image. (*C*) Quantification of dead cells and SA-β-gal positive cells by flow cytometer. *D-F*, Additional data of Fig. 3*F-J*. Cell density was higher than in Fig. 3*F-J*. α-Toc was added to the conditioned medium prepared as in *A*. (*D*) Experimental outline. (*E*) Optical microscope image. (*F*) Quantification of dead cells and SA-β-gal positive cells by flow cytometer. Scale bars in *B* and *K* represent 100 µm. The box and whisker plots of *C* and *F* show the 25th and 75th percentile quartiles and median values (center black line) and maximum and minimum values of the data. *p* value of *C* and *F* by Tukey’s test after two-way ANOVA. *p* values of cell death (%) of *C* and *F* by two-way ANOVA were not significant (respectively Fig. S7*A* and *B*).

**Supplementary Figure S7. The supernatant from ferroptotic cells decreased SA-β-gal of other cells. The effect was not canceled by RNase and Protease.**

*A* and *B*, Additional data of Fig. S6. (*A*) *p* value of Fig. S6*C* by two-way ANOVA. (*B*) *p* value of Fig. S6*F* by two-way ANOVA. *C-E*, After MEFs had been exposed to erastin for 24 h, the supernatant medium was exchanged (erastin was removed). After 3 h, the supernatant medium was collected and administered to new MEFs. At that time, RNase A or Trypsin was added. (*C*) Experimental outline. (*D, E*) Quantification of dead cells and SA-β-gal positive cells by flow cytometer. (*F*), Additional data of Fig. S8. *p* value of Fig. S8*E* by two-way ANOVA. The box and whisker plots of *D* and *E* show the 25th and 75th percentile quartiles and median values (center black line) and maximum and minimum values of the data. *p* value of *D* and *E* by Tukey’s test after two-way ANOVA.

**Figure S8. The supernatant from RSL3-induced ferroptotic cells also increased cell death and decreased SA-β-gal in recipient cells.**

*A-E*, Additional data of Fig. 4*A-E*. Cell density was higher than in Fig. 4*A-E.* After NIH3T3 cells had been exposed to (1S, 3R)-RSL3 for 6 h, the supernatant medium was exchanged (RSL3 was removed). After 3 h, the supernatant medium was collected and administered to recipient NIH3T3 cells. (*A*) Experimental outline. (*B* and *C*) These are data of donor NIH3T3 cells. (*B*) Optical microscope image. (*C*) Quantification of dead cells by flow cytometer. (*D* and *E*) These are data of recipient NIH3T3 cells. (*D*) Optical microscope image. (*E*) Quantification of dead cells and SA-β-gal positive cells by flow cytometer. *F-J*, After MEFs had been exposed to (1S, 3R)-RSL3 for 6 h, the supernatant medium was exchanged (RSL3 was removed). After 3 h, the supernatant medium was collected and administered to recipient MEFs. (*F*) Experimental outline. (*G* and *H*) These are data of donor MEFs. (*G*) Optical microscope image. (*H*) Quantification of dead cells by flow cytometer. (*I* and *J*) These are data of recipient MEFs. (*I*) Optical microscope image. (*J*) Quantification of dead cells and SA-β-gal positive cells by flow cytometer. Scale bars in *B, D, G* and *I* represent 100 µm. The box and whisker plots of *C, E, H,* and *J* show the 25th and 75th percentile quartiles and median values (center black line) and maximum and minimum values of the data. *p* value of *C* and *H* by two-sided *t*-test. *p* value of *E* and *J* by Tukey’s test after two-way ANOVA. *p* values of cell death (%) of *E* by two-way ANOVA were not significant (Fig. S7*F*).

**Supplementary Figure S9. Lipid peroxidation propagated from ferroptotic cells to other cells.**

*A-D*, WT MEFs were exposed to erastin for 24 h. (*A*) Quantification of dead cells by flow cytometer. (*B*) Optical and fluorescent microscope image after Liperfluo staining. (*C*) Representative data of histogram of Liperfluo fluorescence by flow cytometer. The numbers on the upper left and upper right represent respectively the percentage of negative and positive cells. The colors correspond to histogram traces. (*D*) Quantification of Liperfluo positive cells and mean fluorescence intensity (MFI) by flow cytometer. (*E*) Additional data of Fig. 5*A* and *B*. After WT MEFs had been exposed to erastin, the supernatant medium was exchanged (erastin was removed). At that time, Kusabira Orange (KuO) mice-derived MEFs were added. Optical and fluorescent microscope image after Liperfluo staining. White rectangles show the part of Fig. 5*B*. Scale bars in *B* represent 75 µm. Scale bars in *E* represent 250 µm. *A-E* are representative of two independent experiments. Error bars of *A* and *D* represent S.D. *p* value of *A* and *D* by unpaired two-sided *t* test.

**Supplementary Figure S10. Cell death propagated from ferroptotic cells to other cells.**

*A-E*, Additional data of Fig. 5*A-I*. After WT MEFs had been exposed to erastin, the supernatant medium was exchanged (erastin was removed). At that time, KuO mice-derived MEFs were added. (*A*) Representative data of histogram of KuO fluorescence by flow cytometer to distinguish KuO MEFs from WT MEFs. (*B*) Representative data of contour plots of DAPI and AnnexinV fluorescence in WT MEFs by flow cytometer. (*C*) Representative data of contour plots of DAPI and AnnexinV fluorescence in KuO MEFs by flow cytometer. (*D*) Representative data of histogram of KuO fluorescence by flow cytometer. (*E*) Representative data of contour plots of DAPI and AnnexinV fluorescence in KuO MEFs by flow cytometer. Values in each area represent respectively the percentage of cells. DAPI positive or AnnexinV positive cells were judged as dead cells.

**Supplementary Figure S11. The original pictures of Fig. 5 before adjustment**

(*A*) The original pictures of Fig. 5*B* before adjustment. Light green scale bars represent 75 µm. Purple scale bars represent 50 µm.

**Supplementary Figure 12. The original pictures of Fig. S9 before adjustment**

(*A*) The original pictures of Fig. S9*B* before adjustment. (*B*) The original pictures of Fig. S9*E* before adjustment. Scale bars in *A* represent 75 µm. Scale bars in *B* represent 250 µm.
