# Supplementary material for: Lipid peroxidation and the subsequent cell death transmitting from ferroptotic cells to neighboring cells
Source: Cell Death Dis. 2021 Mar 29;12(4):332. doi: 10.1038/s41419-021-03613-y (PMC8007748; doi:10.1038/s41419-021-03613-y)
Supplement: Supplementary file 2 — Supplementary Movie legends [file 41419_2021_3613_MOESM2_ESM.docx]

**Supplementary Movie S1. Cell deaths propagated from ferroptotic cells to surrounding cells.**

*A-F*, After WT MEFs had been exposed to erastin, the supernatant medium was exchanged to Annexin V binding buffer including 10% (v/v) FBS (erastin was removed). At that time, Kusabira Orange (KuO) mice-derived MEFs were added. After that, MEFs were observed by fluorescent microscope for 16 h. (*A* and *B*) Time lapse image of WT MEFs, not exposed to erastin, and KuO MEFs. KuO MEFs were not dying. (*C-F*) Time lapse image of WT MEFs, exposed to 10 µM erastin, and KuO MEFs. (*C*) In the central area, one KuO MEF was dying, attached to a dead WT MEF that was stained with FITC-Annexin V. (*D*) In the central area, one KuO MEF was dying in the vicinity of dead WT MEFs that were stained with FITC-Annexin V. (*E*) In the central area, one KuO MEF was dying surrounded by dead WT MEFs that were stained with FITC-Annexin V. (*F*) In the central area, one WT MEF was dying, shrinking, and being stained with FITC-Annexin V. After that, three KuO MEFs (one is in the upper area, two are in the right side) were dying and finally detached from the bottom of the well.
