# Supplementary material for: Lipid peroxidation and the subsequent cell death transmitting from ferroptotic cells to neighboring cells
Source: Cell Death Dis. 2021 Mar 29;12(4):332. doi: 10.1038/s41419-021-03613-y (PMC8007748; doi:10.1038/s41419-021-03613-y)
Supplement: Supplementary file 3 — Supplementary Tables [file 41419_2021_3613_MOESM3_ESM.pdf]

Supplementary Table S1. siRNA

siRNA	Sequence
si*Atg7* #1	5'-CAAUGAUGUGGUGGCUCCAGGAGAU-3'
si*Atg7* #2	5'-CAGCCUGGCAUUUGAUAAAUGUACA-3'

Supplementary Table S2. qPCR primers

Gene	Forward primer	Reverse primer
Actb	5'-CGTTGACATCCGTAAAGACCTC-3'	5'-AGCCACCGATCCACACAGA-3'
Atg7	5'-TATGGACCCCAAAAGGCTGG-3'	5'-AGCCACATTACACCCCAAGG-3'
Glb1	5'-CGGATACCCCGCTTCTACTG-3'	5'-GGCACGTACATCTGGATAGC-3'
