# Supplementary figures and images for: Lipid peroxidation and the subsequent cell death transmitting from ferroptotic cells to neighboring cells
Source: Cell Death Dis. 2021 Mar 29;12(4):332. doi: 10.1038/s41419-021-03613-y (PMC8007748; doi:10.1038/s41419-021-03613-y)

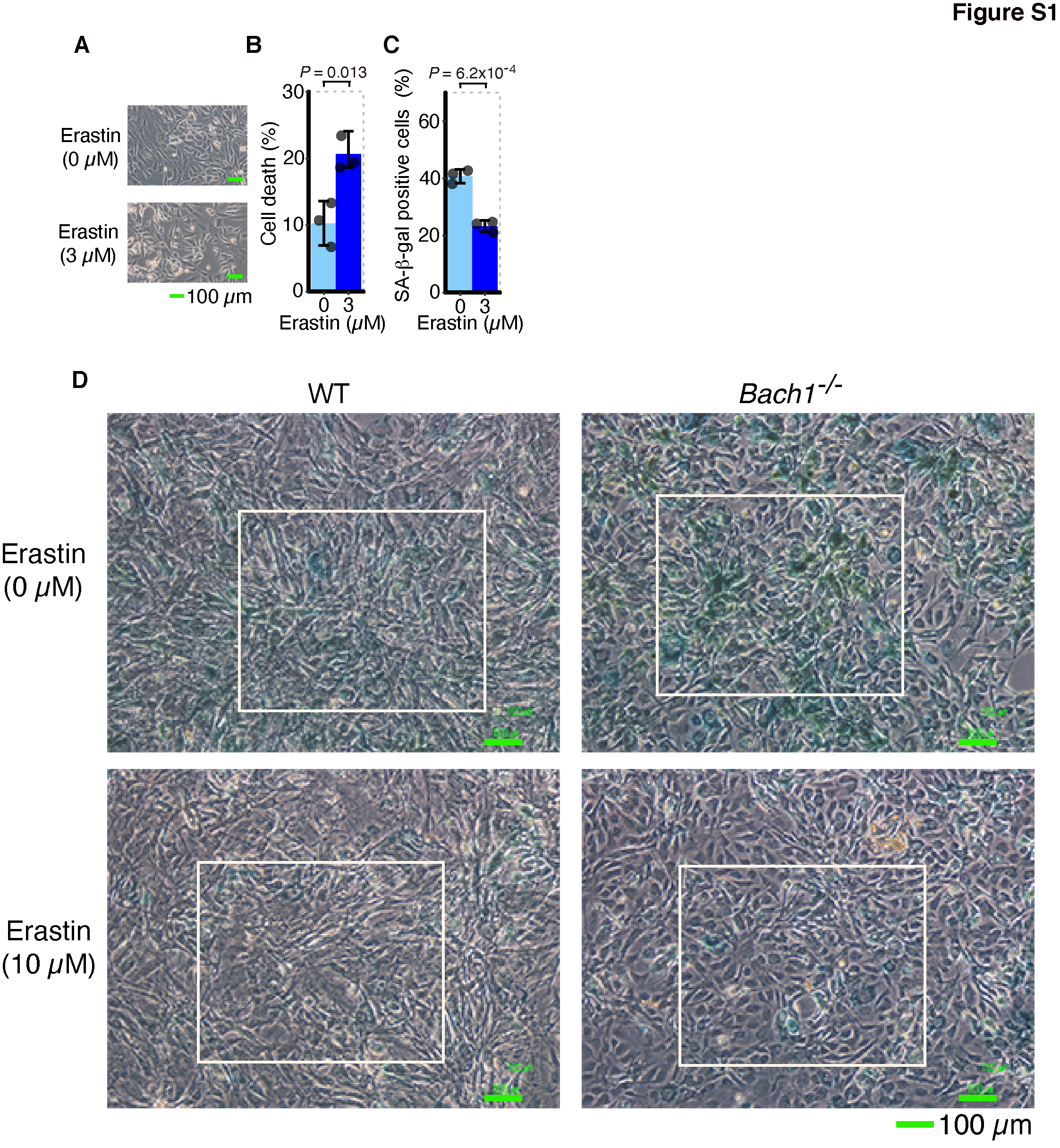

Supplement: Supplementary file 4 — Supplementary Figure S1 [file 41419_2021_3613_MOESM4_ESM.png]

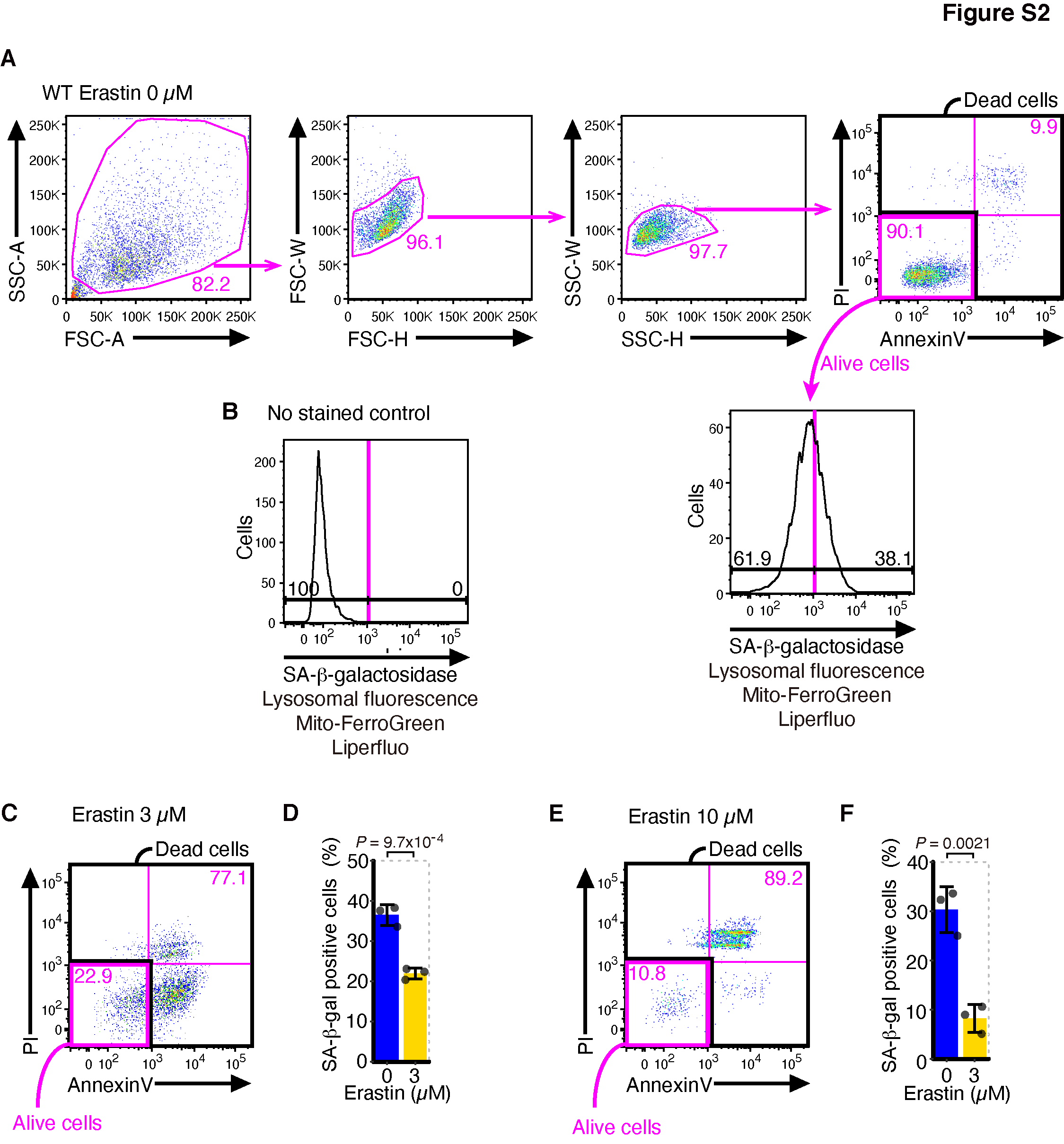

Supplement: Supplementary file 5 — Supplementary Figure S2 [file 41419_2021_3613_MOESM5_ESM.png]

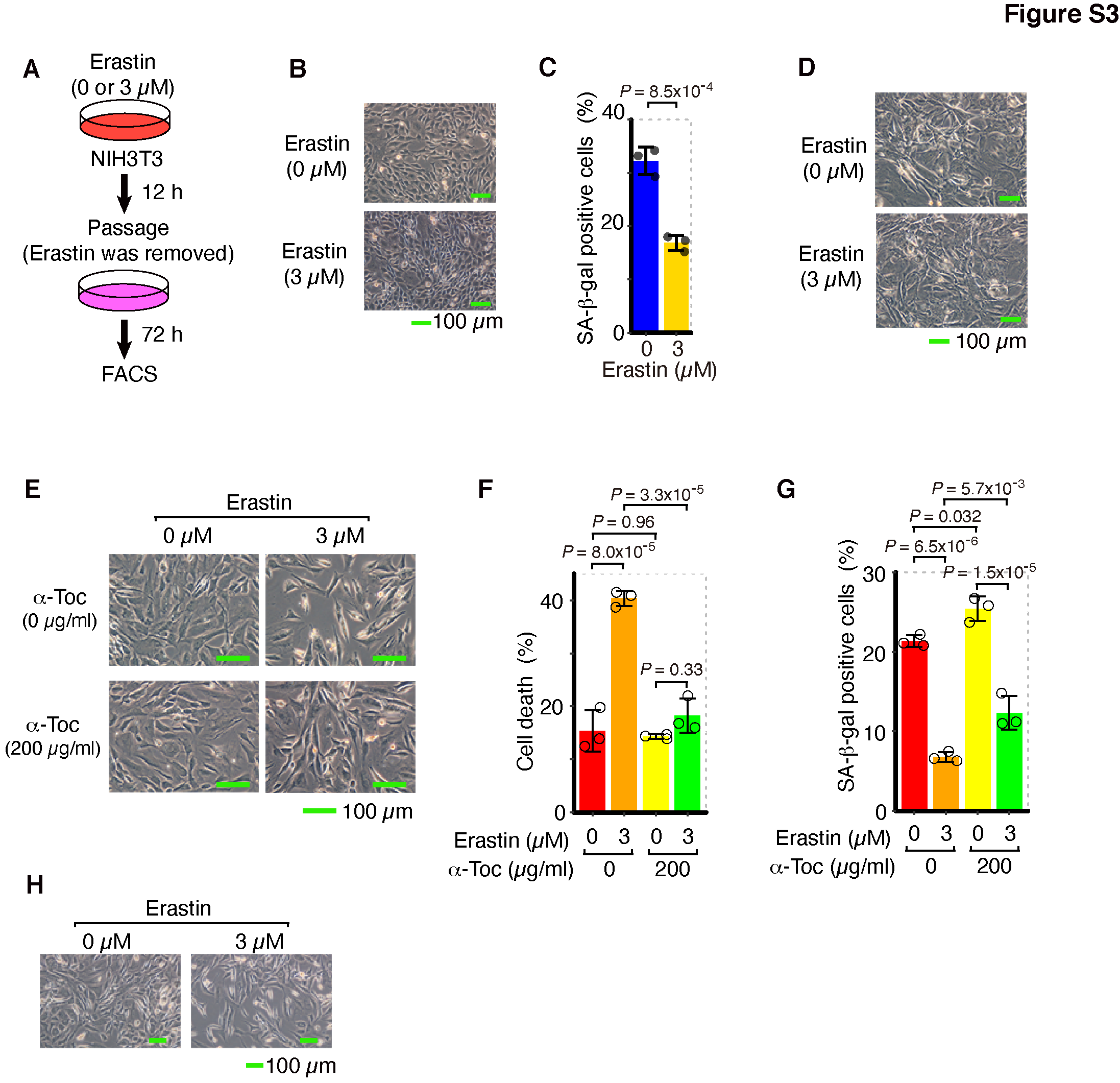

Supplement: Supplementary file 6 — Supplementary Figure S3 [file 41419_2021_3613_MOESM6_ESM.png]

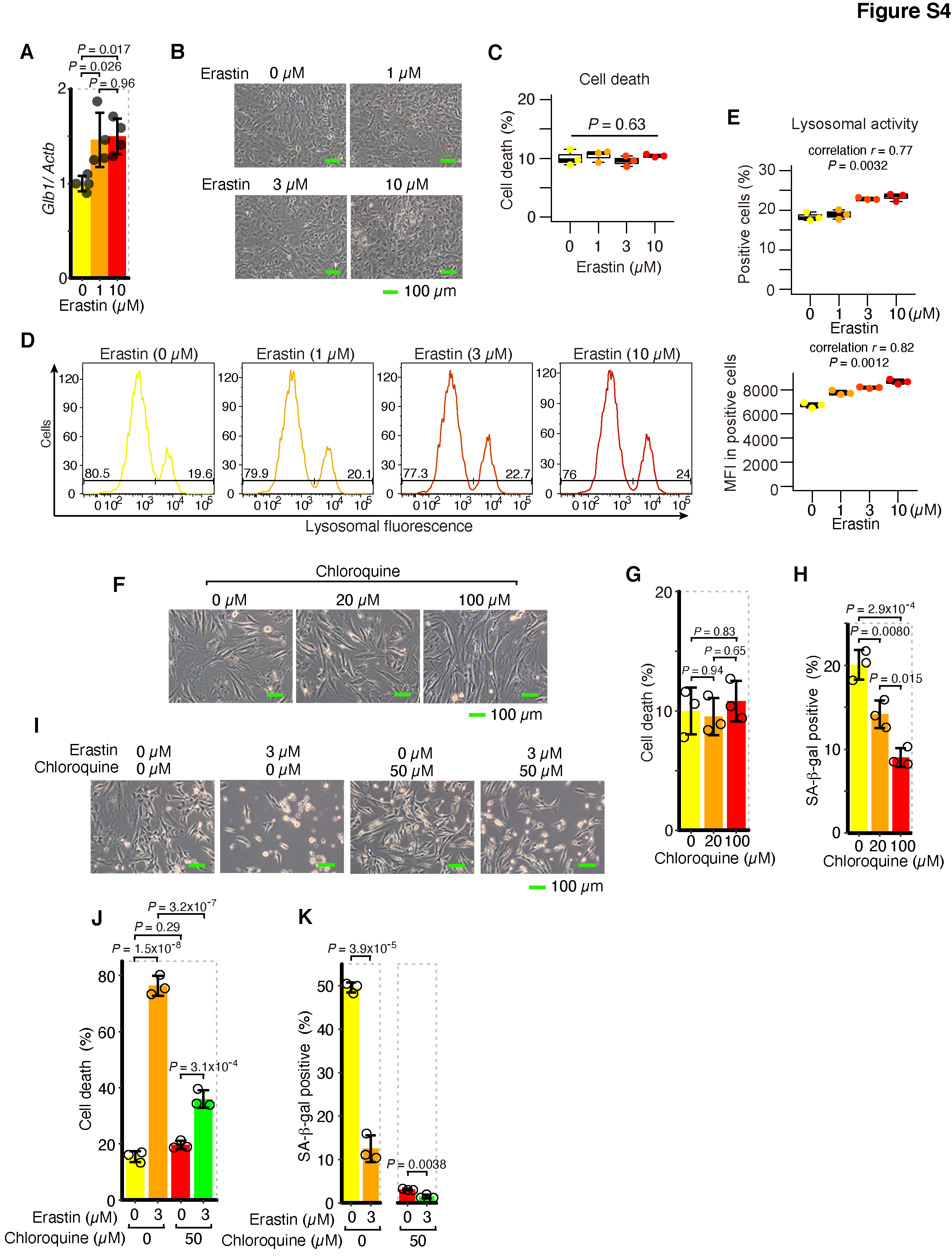

Supplement: Supplementary file 7 — Supplementary Figure S4 [file 41419_2021_3613_MOESM7_ESM.png]

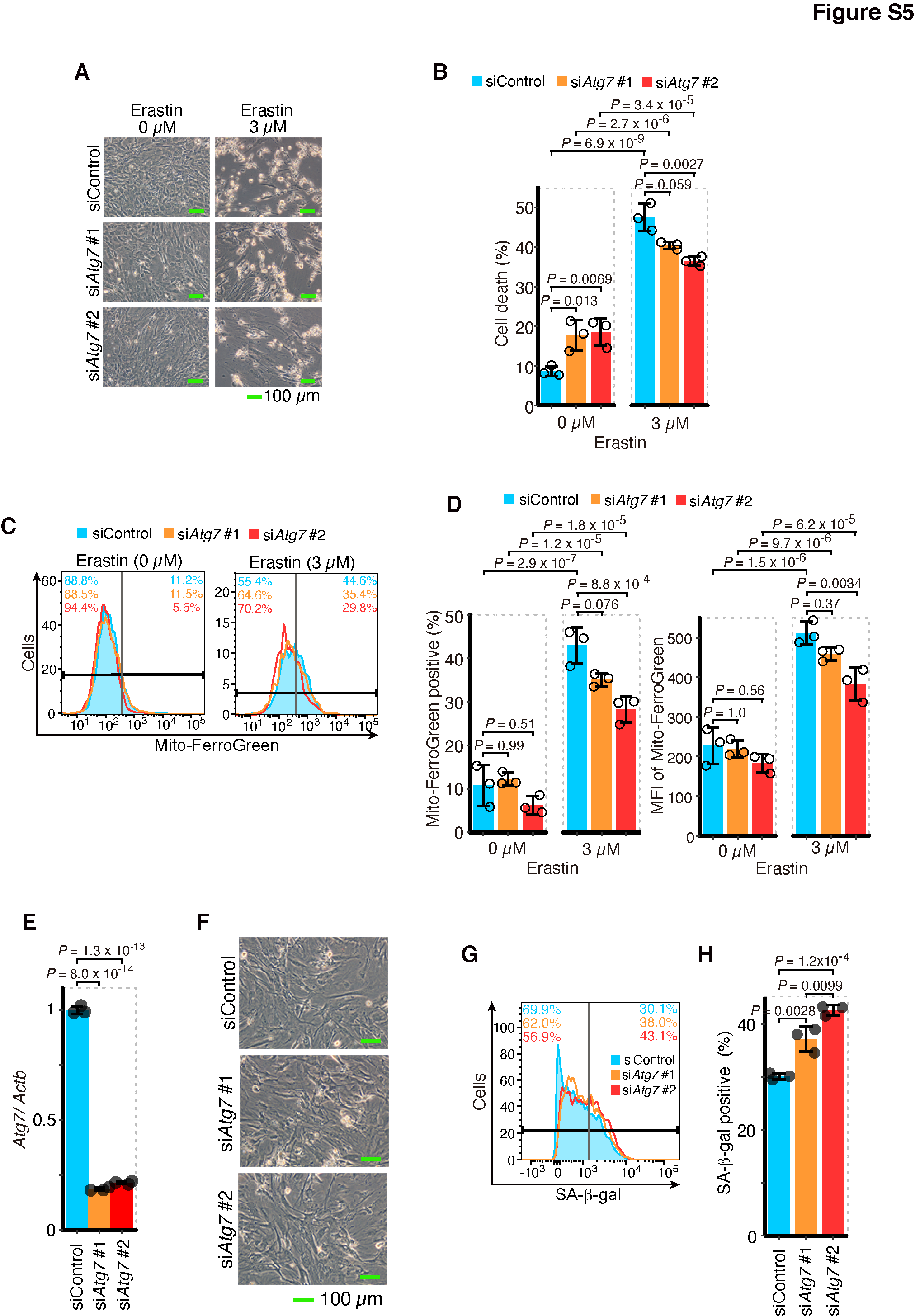

Supplement: Supplementary file 8 — Supplementary Figure S5 [file 41419_2021_3613_MOESM8_ESM.png]

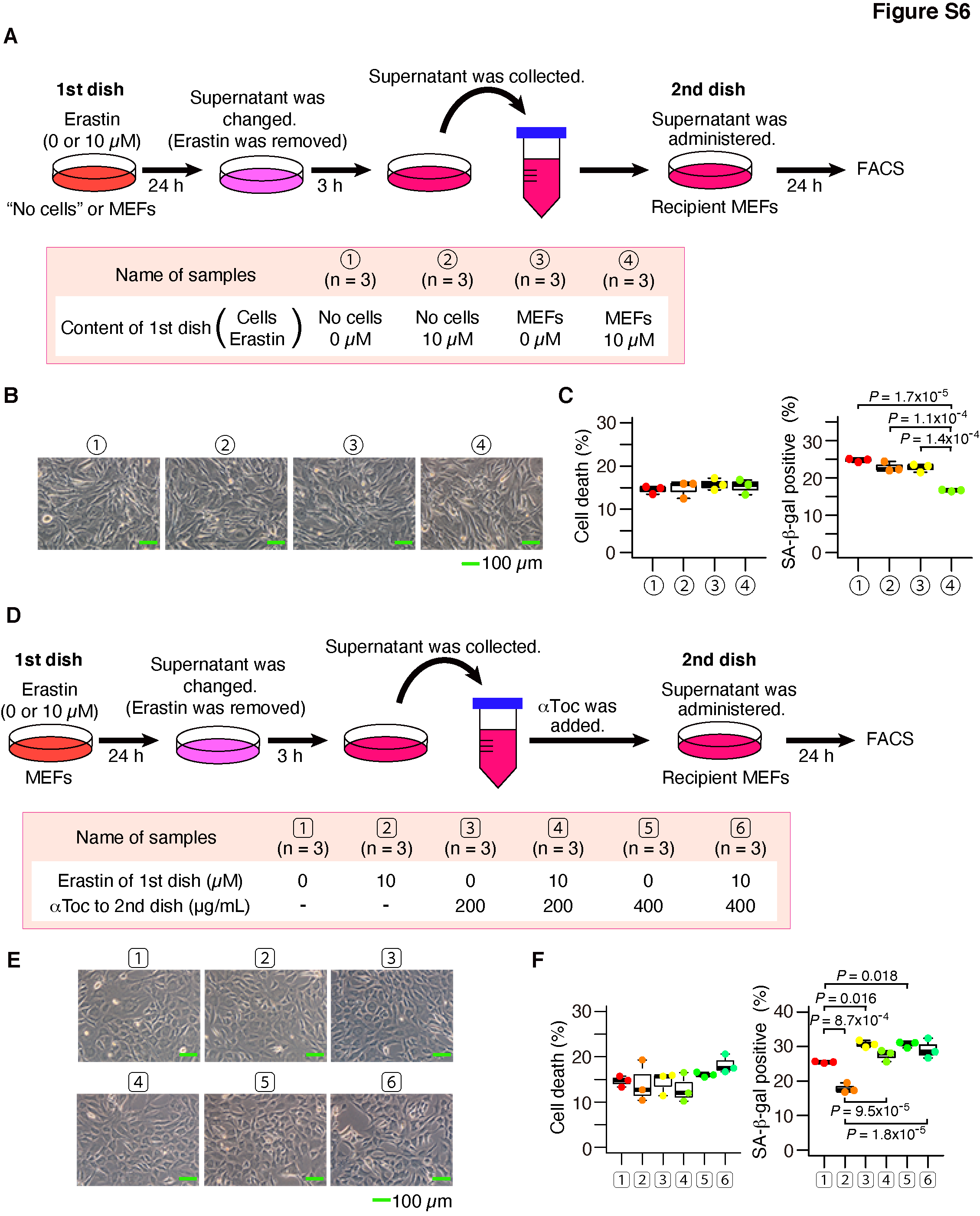

Supplement: Supplementary file 9 — Supplementary Figure S6 [file 41419_2021_3613_MOESM9_ESM.png]

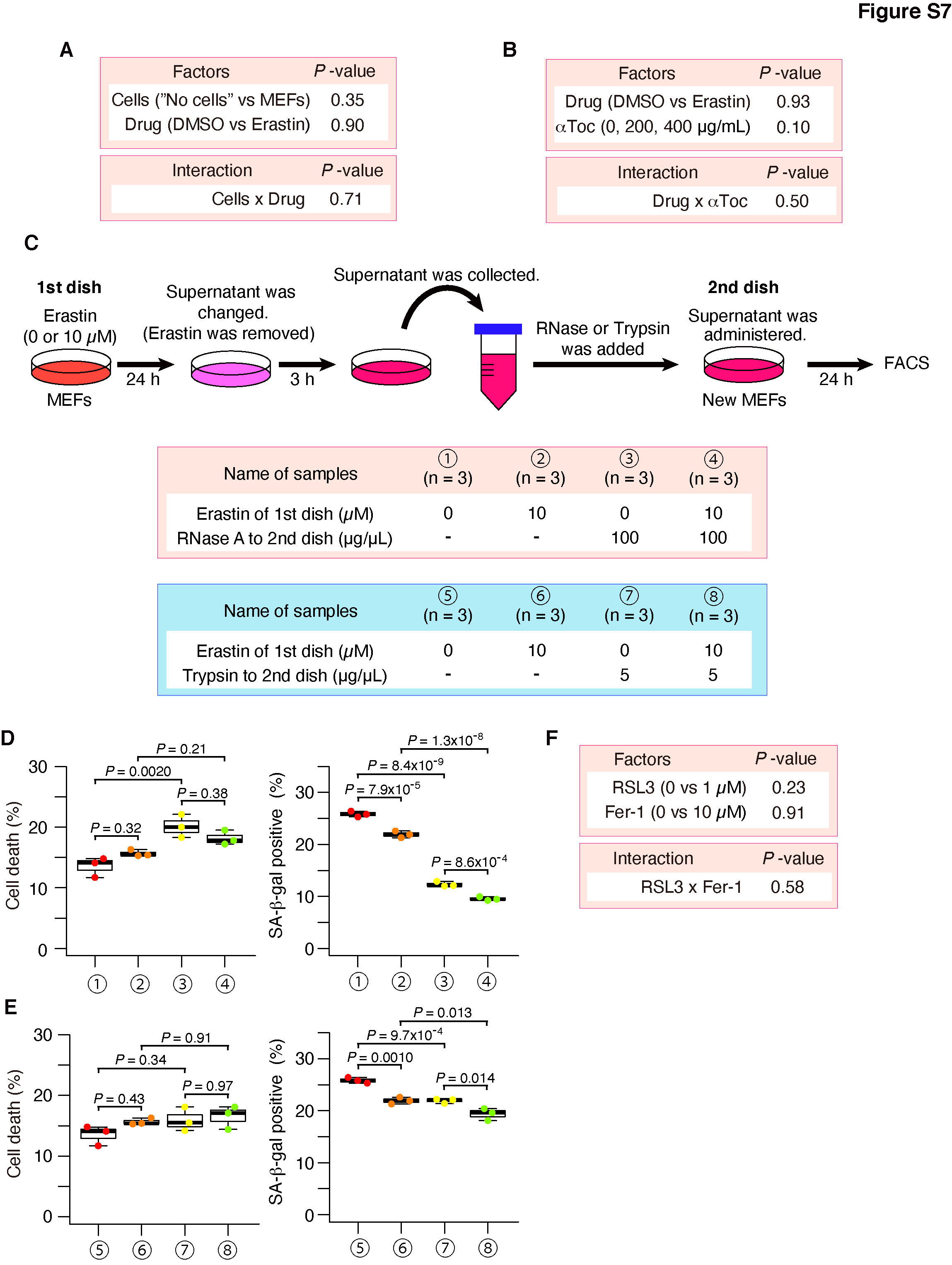

Supplement: Supplementary file 10 — Supplementary Figure S7 [file 41419_2021_3613_MOESM10_ESM.png]

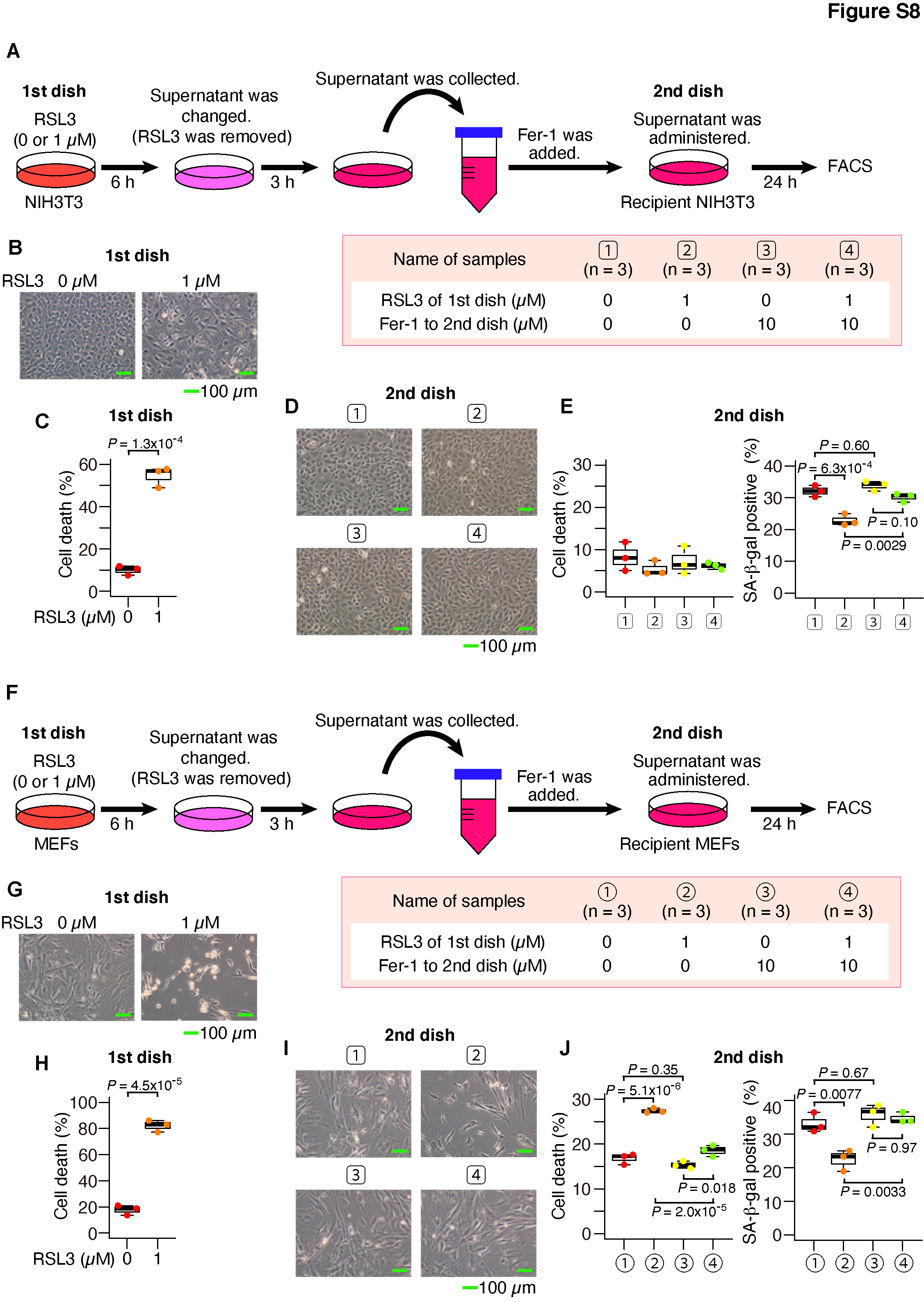

Supplement: Supplementary file 11 — Supplementary Figure S8 [file 41419_2021_3613_MOESM11_ESM.png]

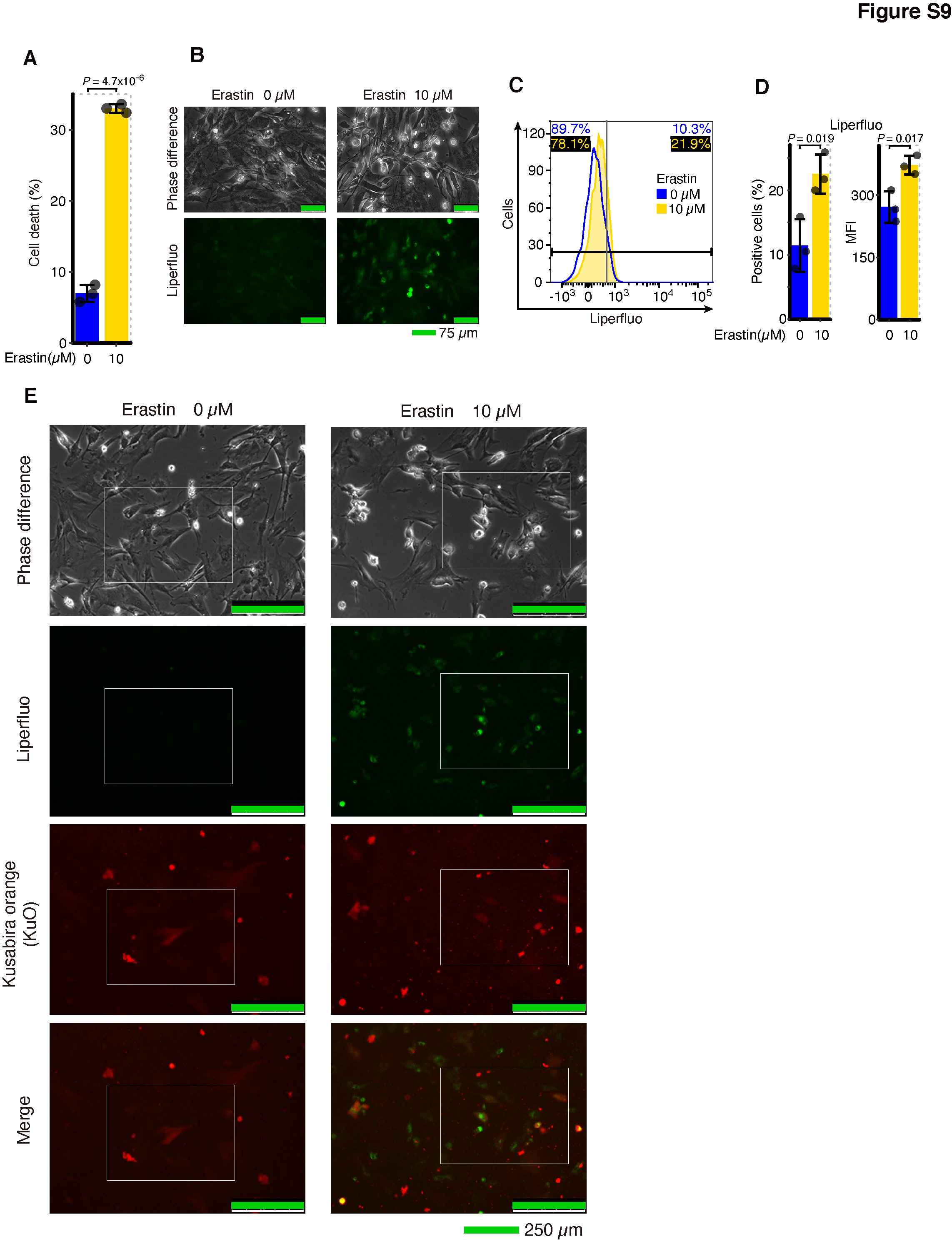

Supplement: Supplementary file 12 — Supplementary Figure S9 [file 41419_2021_3613_MOESM12_ESM.png]

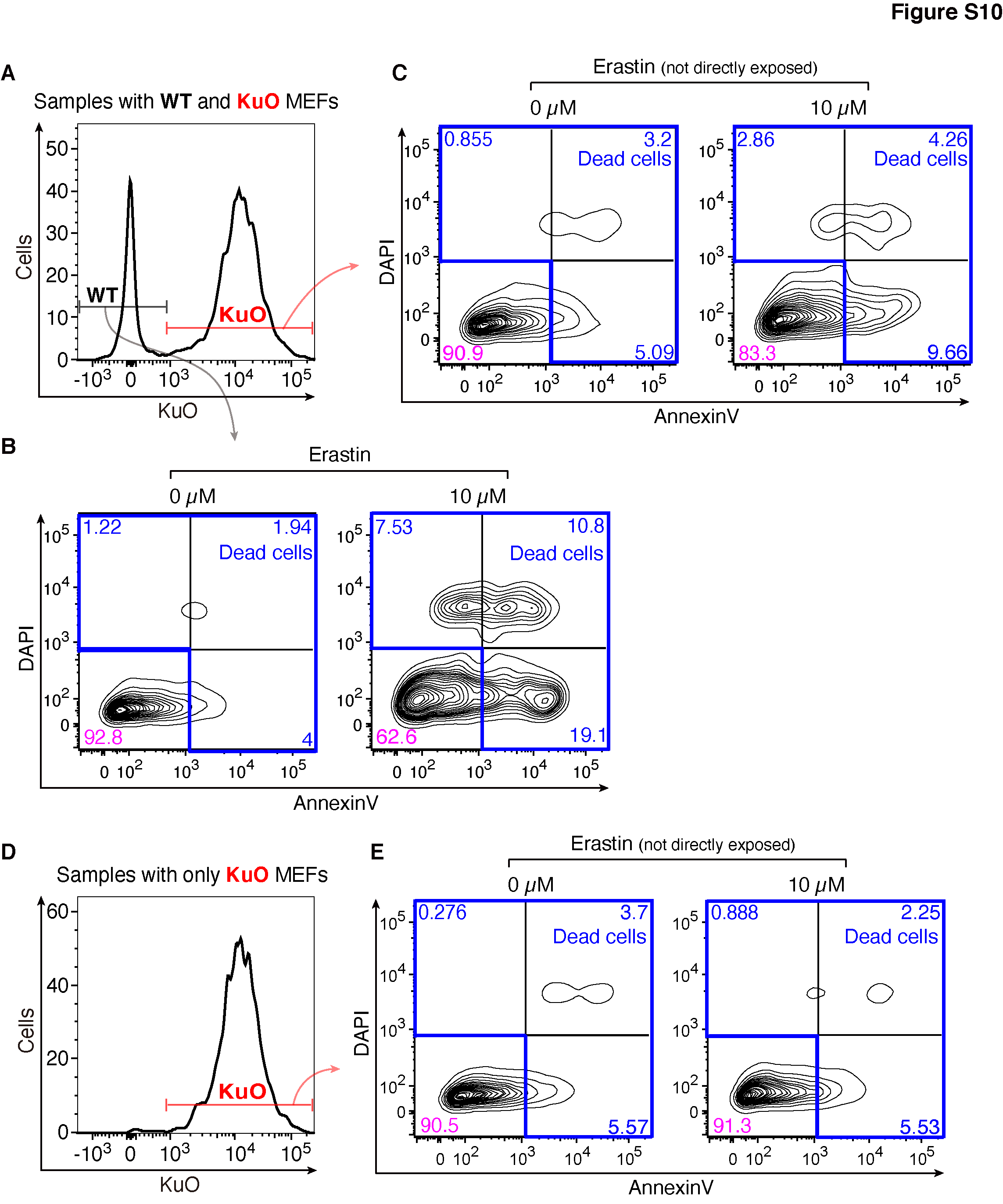

Supplement: Supplementary file 13 — Supplementary Figure S10 [file 41419_2021_3613_MOESM13_ESM.png]

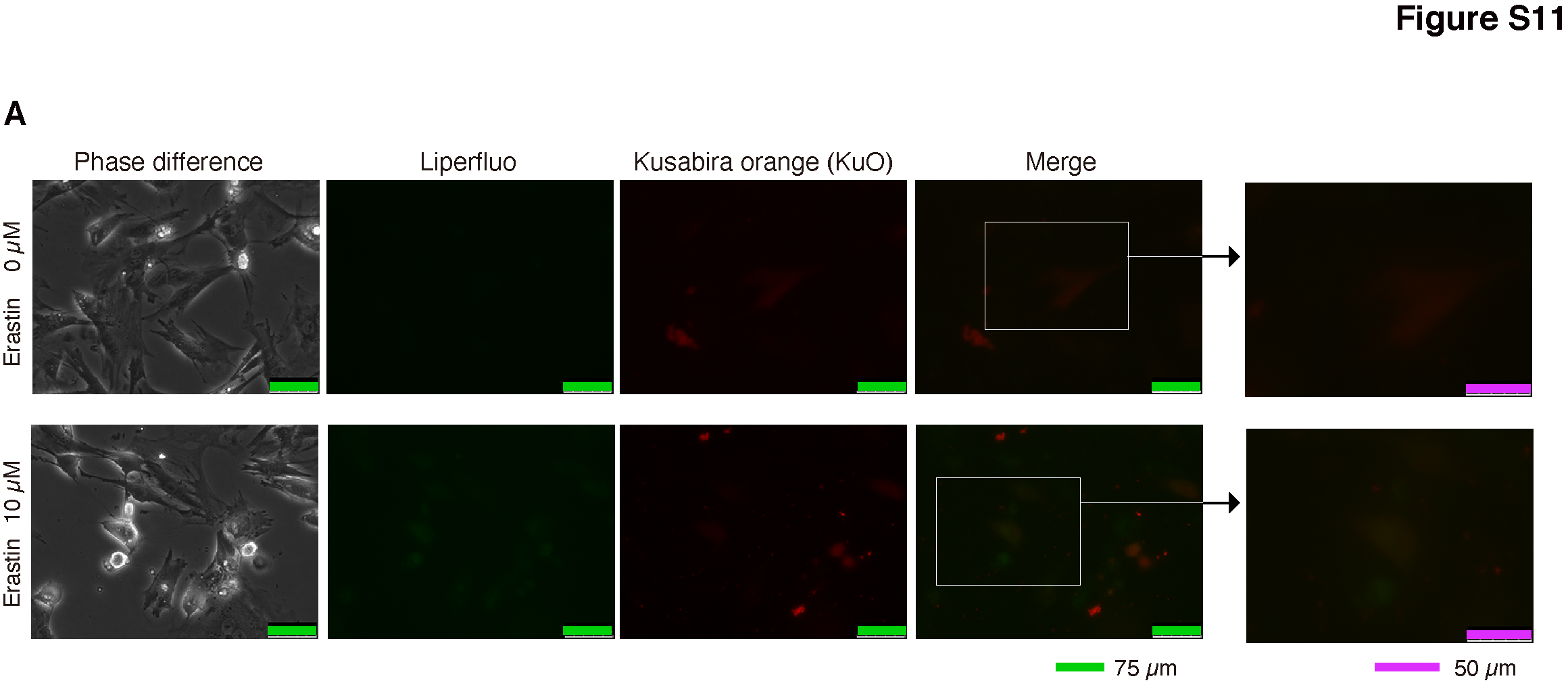

Supplement: Supplementary file 14 — Supplementary Figure S11 [file 41419_2021_3613_MOESM14_ESM.png]

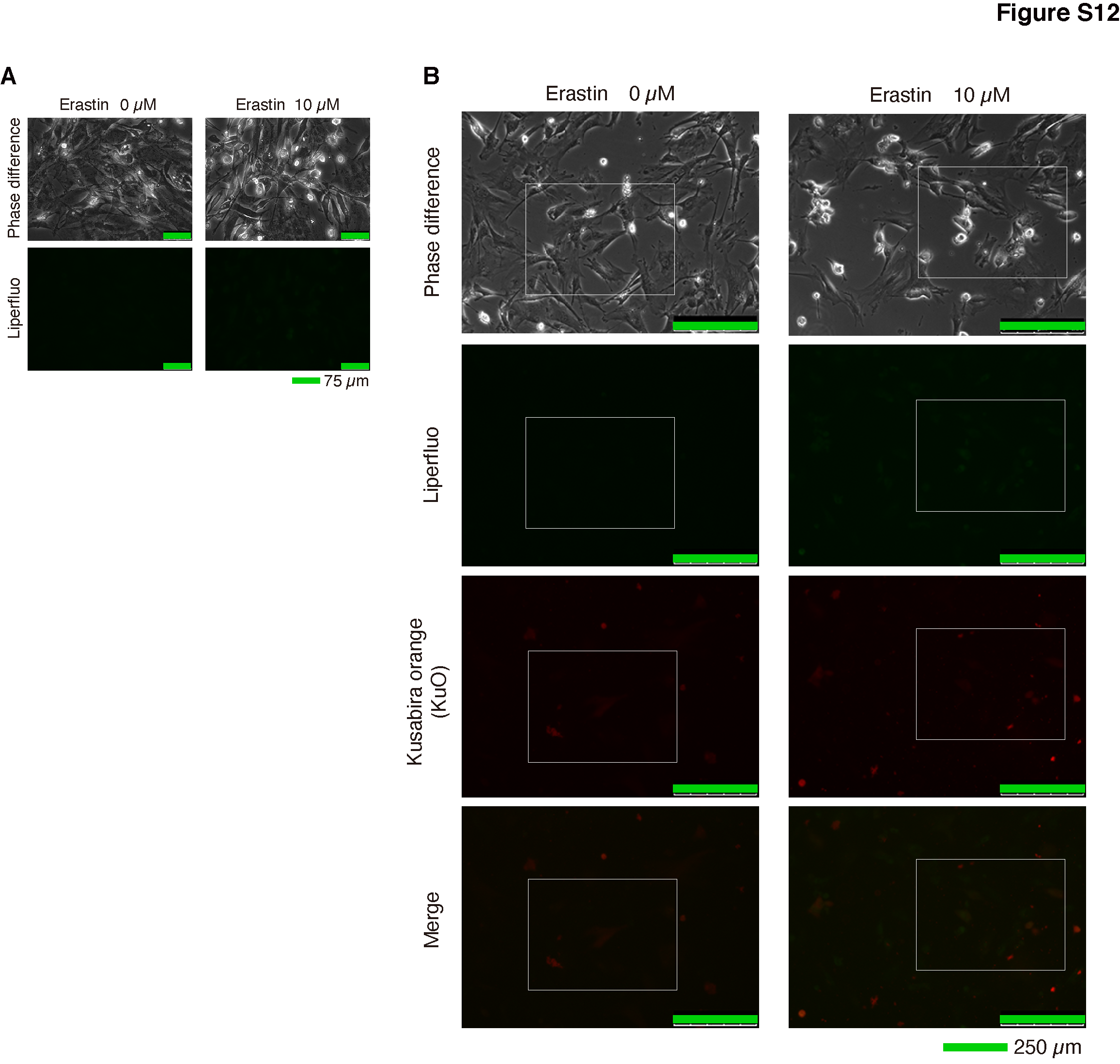

Supplement: Supplementary file 15 — Supplementary Figure S12 [file 41419_2021_3613_MOESM15_ESM.png]
